# Prostate specific antigen retesting intervals and trends in England: population based cohort study

**DOI:** 10.1136/bmj-2024-083800

**Published:** 2025-10-08

**Authors:** Kiana K Collins, Jason L Oke, Pradeep S Virdee, Rafael Perera, Brian D Nicholson

**Affiliations:** Nuffield Department of Primary Care Health Sciences, University of Oxford, Oxford OX2 6GG, UK

## Abstract

**Objective:**

To characterise the use of the prostate specific antigen (PSA) test in primary care in England.

**Design:**

Population based open cohort study.

**Setting:**

England.

**Participants:**

10 235 805 male patients older than 18 years and registered at 1442 general practices that contributed to the Clinical Practice Research Datalink between 2000 and 2018. Data were linked to the National Cancer Registry, Hospital Episode Statistics, and Office for National Statistics.

**Main outcome measures:**

Population based temporal trends and annual percentage changes were analysed using age standardised PSA testing rates. Mixed effects negative binomial regression models investigated individual patient rate ratios of PSA testing. Linear mixed effects models examined factors associated with an individual patient’s length of PSA retesting intervals. All results were analysed by region, deprivation, age, ethnicity, family history of prostate cancer, symptom presentation, and PSA value.

**Results:**

1 521 116 patients had at least one PSA test, resulting in 3 835 440 PSA tests overall. 48.4% (735 750) of these patients had multiple tests and 72.8% (535 990) of them never presented with a PSA value above the age specific referral threshold. The median retesting interval overall was 12.6 months (interquartile range 6.2-27.5). Testing rates varied by region, deprivation, ethnicity, family history, age, PSA value, and symptoms. Once tested, patients had shorter retesting intervals if they were older, were of an ethnicity other than white, had a family history of prostate cancer, or had previously raised PSA levels. Despite considerable variation in testing rates by region and deprivation, the length of retesting intervals was similar across these groups.

**Conclusions:**

PSA testing before a diagnosis of prostate cancer in primary care in England varied. Among patients who underwent multiple tests, many were tested more frequently than recommended, raising concerns about overtesting. PSA retesting is occurring in patients without recorded symptoms and in those with low PSA values. To ensure maximum benefit to patients while reducing the risk of overtesting, research is urgently needed to determine appropriate evidence based PSA retesting intervals.

## Introduction

Prostate cancer is the most common cancer diagnosed in the UK.^1^ The prostate specific antigen (PSA) test is the initial triage step for patients with symptoms presenting to primary care in England. The National Institute for Health and Care Excellence (NICE) NG12 guidelines were developed to support primary care professionals in the recognition and referral of people with suspected cancer in England and Wales. The guidelines recommend considering PSA testing for patients who present with lower urinary tract symptoms, erectile dysfunction, or visible haematuria.^2^ If a patient’s PSA value is above an age specific threshold, referral to secondary care is advised. NICE NG12 does not provide a recommended interval for PSA retesting, nor does it specify age thresholds to start or stop testing. It is possible that patients are retested if symptoms persist.^3^

PSA testing recommendations in primary care are different for asymptomatic patients. No international guidelines currently endorse routine population wide PSA screening.^4^ Lithuania has a PSA testing programme every two years for asymptomatic patients, but only for those attending primary care for another reason.^5^ The UK National Screening Committee recommends against PSA screening because although screening with PSA reduces prostate cancer specific mortality, its effect on overall mortality is small^6^ ^7^ and it is unclear whether the benefits from screening outweigh the harms of overdiagnosis, overtreatment, or uncertain findings.^8^ In England, however, the Prostate Cancer Risk Management Programme (PCRMP) advises that men older than 50 years can request a PSA test if they make a shared decision with their clinician. If the patient’s PSA result is ≥3 ng/mL, the PCRMP recommends referral to secondary care.^9^ PCRMP does not provide a recommended interval for retesting, or the age at which testing should stop.^9^ Conflicting guidance in the UK makes it difficult to discern whether PSA testing is encouraged or discouraged.

Internationally, the recommended interval to retest PSA ranges from one year to 10 years.^10^ This variation in clinical guidelines reflects the lack of direct evidence for PSA retesting intervals. Reasons for retesting in primary care are largely unknown, other than to confirm a raised PSA value.^11^ Repeating a test for asymptomatic patients as part of routine annual screening increases the risk of overdiagnosis and overtreatment of prostate cancer.^4^ ^12^

PSA testing in the UK has been evaluated in several cross sectional^13^ ^14^ and longitudinal studies.^15^ However, these studies had short timeframes or used survival analysis methods focused on the risk of having one PSA test. Increased rates of testing over time and inequalities in the UK around the cumulative risk of having a PSA test have been shown.^13^ ^14^ ^15^ It has been established that PSA testing is higher in the south of England and in areas of lower deprivation.^4^ Patterns for retesting intervals, rates of testing by PSA result, and the impact of symptom presentation in primary care are unknown.

In this study, we characterised how PSA testing was utilised in primary care between 2000 and 2018. Our objectives were to describe population based trends in PSA testing over time and to examine individual patient variation in both the rates of PSA testing and the length of PSA retesting intervals. We evaluated associations with region, deprivation, ethnicity, age, family history of prostate cancer, PSA test results, and symptom presentation to explore whether variation in overall testing rates was driven by differences in how frequently patients were retested.

## Methods

### Study population

We conducted an open, population based cohort study of routinely collected data in electronic health records from the Clinical Practice Research Datalink (CPRD) Aurum linked to the National Cancer Registration and Analysis Service (NCRAS), Hospital Episodes Statistics (HES), and the Office for National Statistics (ONS) death registration data. The study period was between 1 January 2000 and 31 December 2018 and is a part of the approved CPRD protocol (ID: 22_001798) designed to investigate the role of blood test trends for cancer detection in patients attending primary care.^16^ All results are reported according to the REporting of studies Conducted using Observational Routinely Collected health Data (RECORD) extension to the Strengthening the Reporting of Observational Studies in Epidemiology (STROBE) guidelines for observational studies.

Eligible male patients were registered at English general practices between 2000 and 2018, linkable to NCRAS, HES, and ONS, contributed at least one year of follow-up, did not have a prostate cancer diagnosis before entering the study, and were older than 18 years during the study period. Patients entered the cohort at the latest of date of the start of the study (1 January 2000), registration with the practice, or 18th birthday. Patients exited the cohort at the earliest date of study end (31 December 2018), first prostate cancer diagnosis, death, transference out of the practice, or last data download for that practice.

### Variables

We excluded PSA tests for all patients that occurred before patients entered the cohort or after patients exited the cohort. For patients with a prostate cancer diagnosis during follow-up, we included PSA tests occurring before the patient’s earliest cancer diagnosis date. Each PSA test record was defined as being either above or below an age specific threshold. This was based on the NICE NG12 age specific PSA thresholds for patients with symptoms of prostate cancer.^2^ PSA values were categorised as above the age specific threshold if the patient was aged 18-49 years with a PSA value >2.5 ng/mL, aged 50-59 years with a PSA value >3.5 ng/mL, aged 60-69 years with a PSA value >4.5 ng/mL, and aged ≥70 years with a PSA value >6.5 ng/mL.

We included seven symptom categories on the basis of NICE guidelines NG12^2^: back pain, bone pain, fatigue, weight loss, haematuria, erectile dysfunction, and lower urinary tract symptoms. Symptoms were retrieved from the patients’ primary care record (CPRD Aurum) based on SNOMED-CT codes. We paired PSA tests with a symptom if the symptom occurred in the 90 days before the test date. If PSA tests occurred in the first three months of 2000, we retrieved symptoms occurring in the previous 90 days.

Ethnicity was grouped into white, Asian, black, South Asian, mixed, other, and unknown. Supplementary file 1 provides further details on curation of variables. A clinician (BDN) validated all variable code lists (see supplementary file 4).

### Patient characteristics

Individual patient characteristics determined at the date of study entry were age group (18-29, 30-39, 40-49, 50-59, 60-69, 70-79, 80-89, ≥90 years), ethnicity, family history of prostate cancer, whether the patient had a diagnosis of prostate cancer during follow-up, region, and index of multiple deprivation fifth.

#### PSA testing trends overtime

Population based crude PSA testing rates were calculated by dividing the number of tests ordered per 1000 person years of follow-up overall and by year. Age standardised testing rates were estimated using direct standardisation, with 2018 as the reference population. Temporal trends of PSA testing rates between 2000 and 2018 were stratified by age at PSA test, region, index of multiple deprivation fifth, ethnic group, family history, PSA value above or below the age specific threshold, and presence of at least one symptom paired with a PSA test. We did not replace any missing data as we aimed to describe patterns of PSA testing recorded in primary care.

#### PSA testing rates

To model individual PSA testing rates, we used univariate and multivariable mixed effects negative binomial regression models with an offset term for the log of person years of follow-up. Fixed effects included age range, ethnicity, index of multiple deprivation, region, family history of prostate cancer, if the patient ever had a symptom paired with a PSA test, and if the patient ever had a PSA value above the age specific threshold. A random intercept for general practice was included to account for clustering at the practice level. We obtained rate ratios for the number of PSA tests by exponentiating the model coefficients.

#### PSA retesting intervals

**Fig 1:**
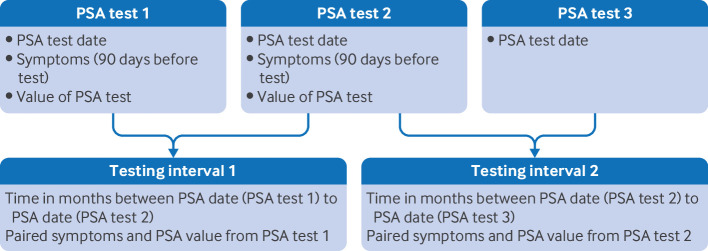
Construction of PSA retesting intervals. Symptoms and PSA values for interval are taken from the earlier test (the PSA value at that test and symptoms recorded in the preceding 90 days). PSA=prostate specific antigen

We used descriptive statistics to summarise PSA retesting intervals in months. If a patient had three PSA tests they contributed two intervals. We used symptoms and PSA value paired with the PSA test at the start of the interval. The patient’s last recorded PSA test result and associated symptoms were not included in the interval calculation because details of subsequent testing were unknown (fig 1).

We used univariate and multivariable linear mixed effects regression models to identify patient factors associated with the log transformed length of the PSA retesting interval (in months). Nested random intercepts for patients within practices accounted for both the non-independence of repeated measures within patients and the clustering within general practices. Fixed effects were the same as above. Expected months between PSA tests were calculated by exponentiating the model intercept, representing the log geometric mean interval for the reference group and multiplying this by the exponentiated fixed effect for each covariate level.

We estimated 95% confidence intervals (CIs), and a two sided significance level of 5% was used for all analyses. Characteristics used as the reference group were age 60-69 years, white ethnicity, first fifth of index of multiple deprivation (least deprived), no family history of prostate cancer, no symptom paired with a PSA test, and never had a PSA value above the age specific threshold.

#### Subgroup and sensitivity analyses

A subgroup analysis was done to investigate associations between the length of PSA retesting intervals in patients without prostate cancer who had multiple tests but never had a PSA value above the age specific threshold.

Sensitivity analyses were done for two reasons. Firstly, to assess the impact on PSA retesting intervals when only including tests that were more than one month apart, to remove bias from having a repeat PSA test to confirm first raised PSA. Secondly, to assess the impact of restricting to patients with more than six years of follow-up to mitigate potential censoring bias. We analysed cohorts who had more than six years of follow-up from study entry to exit. Sensitivity analyses are included in supplementary file 3.

### Patient and public involvement

A dedicated patient advisory group advised on which analyses are important to patients. They helped with refining and applying the patients’ perspectives to the research findings and reviewed the manuscript.

## Results

A total of 10 235 805 male patients from 1442 practices contributed 81 742 938 person years of observation (table 1). Median follow-up was 5.9 years (IQR 2.9-12.4), with a maximum of 19 years. Overall, 1 521 116 patients had at least one PSA test, and 3 835 440 PSA tests were done overall. Nineteen per cent of the 3 835 440 PSA tests were in patients aged 50-59 years, 31.0% (1 187 800) in those aged 60-69 years, 28.1% (1 076 797) in those aged 70-79 years, and 12.2% (467 291) in those aged 80-89 years. In total, 48.4% (735 750/1 521 116) of patients had at least two PSA tests recorded in the study period (table 1). 

**Table 1:** Patient characteristics. Values are number (percentage) unless stated otherwise

	Overall study cohort (n=10 235 805)	At least 1 PSA test (n=1 521 116)	At least 2 PSA tests (n=735 750)	Subgroup analysis* (n=535 990)
No of practices	1442	1441	1438	1438
Median (IQR) follow-up (years)	5.9 (2.9-12.4)	14.3 (7.9-19.0)	16.4 (10.4-19.0)	17.2 (11.1-19.0)
Person years of observation	81 742 938	19 843 625	10 557 563	7 899 475
Ethnicity:				
White	7 019 072 (68.6)	1 289 012 (84.7)	641 174 (87.1)	463 142 (86.4)
Black	402 798 (3.9)	51 383 (3.4)	23 653 (3.2)	17 071 (3.2)
Asian	304 061 (3.0)	20 068 (1.3)	8848 (1.2)	7225 (1.3)
Mixed	118 876 (1.2)	8542 (0.6)	3447 (0.5)	2617 (0.5)
South Asian	478 353 (4.7)	46 970 (3.1)	21 586 (2.9)	18 180 (3.4)
Other	264 970 (2.6)	21 982 (1.4)	9236 (1.3)	7330 (1.4)
Unknown	1 647 675 (16.1)	83 159 (5.5)	27 806 (3.8)	20 425 (3.8)
Region:				
East Midlands	302 747 (3.0)	38 485 (2.5)	18 725 (2.5)	13 501 (2.5)
East of England	404 147 (3.9)	75 548 (5.0)	37 425 (5.1)	26 933 (5.0)
London	2 366 313 (23.1)	252 147 (16.6)	115 415 (15.7)	87 594 (16.3)
North East	291 735 (2.9)	39 887 (2.6)	16 564 (2.3)	11 957 (2.2)
North West	1 700 935 (16.6)	270 221 (17.8)	131 290 (17.8)	96 860 (18.1)
South East	2 055 582 (20.1)	346 579 (22.8)	174 295 (23.7)	126 795 (23.7)
South West	1 202 150 (11.7)	188 471(12.4)	91 470 (12.4)	63 080 (11.8)
West Midlands	1 526 812 (14.9)	258 948 (17.0)	126 852 (17.2)	92 260 (17.2)
Yorkshire and the Humber	377 935 (3.7)	50 358 (3.3)	23 575 (3.2)	16 917 (3.2)
Unknown	7449 (0.1)	472 (0.0)	139 (0.0)	93 (0.0)
Index of multiple deprivation:				
1 (least deprived/most affluent)	1 930 388 (18.9)	372 309 (24.5)	193 930 (26.4)	139 142 (26.0)
2	2 026 539 (19.8)	344 821 (22.7)	173 569 (23.6)	125 612 (23.4)
3	2 035 452 (19.9)	301 805 (19.8)	146 258 (19.9)	106 207 (19.8)
4	2 244 264 (21.9)	273 790 (18.0)	124 324 (16.9)	91 717 (17.1)
5 (most deprived/least affluent)	1 986 414 (19.4)	226 883 (14.9)	96 972 (13.2)	72 795 (13.6)
Unknown/Missing	12 748 (0.1)	1508 (0.1)	697 (0.1)	517 (0.1)
Age at study entry (years):				
18-29	3 853 853 (37.7)	37 778 (2.5)	5124 (0.7)	4591 (0.9)
30-39	2 328 096 (22.7)	169 384 (11.1)	47 111 (6.4)	40 827 (7.6)
40-49	1 522 747 (14.9)	322 808 (21.2)	136 510 (18.6)	109 803 (20.5)
50-59	1 105 127 (10.8)	406 124 (26.7)	220 923 (30.0)	160 777 (30.0)
60-69	746 148 (7.3)	336 184 (22.1)	197 430 (26.8)	136 534 (25.5)
70-79	461 472 (4.5)	191 741 (12.6)	104 780 (14.2)	69 237 (12.9)
80-89	188 368 (1.8)	52 777 (3.5)	22 632 (3.1)	13 564 (2.5)
≥90	29 994 (0.3)	4320 (0.3)	1240 (0.2)	657 (0.1)
Family history of prostate cancer	34 200 (0.3)	19 245 (1.3)	11 337 (1.5)	8430 (1.6)

IQR=interquartile range; PSA=prostate specific antigen.

* Patients who never had PSA values above the age specific threshold and who did not have a diagnosis of prostate cancer during follow-up. Above the threshold if aged 18-49 years with a PSA value >2.5 ng/mL, aged 50-59 years with a PSA value >3.5 ng/mL, aged 60-69 years with a PSA value >4.5 ng/mL, and aged ≥70 years with a PSA value >6.5 ng/mL.

### PSA testing trends over time at population level

**Fig 2:**
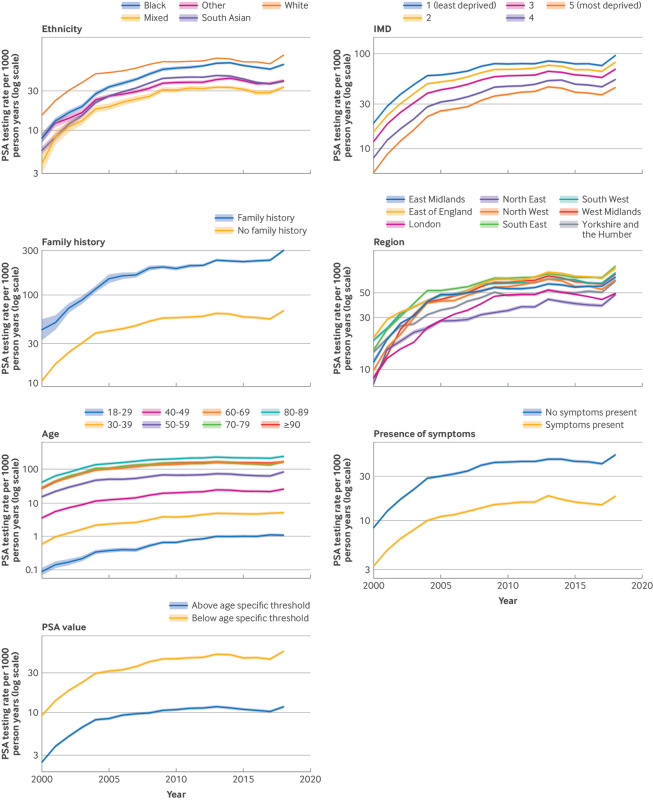
Age standardised PSA testing rates per 1000 person years by patient from 2000 to 2018. Rates are shown with 95% confidence intervals (shaded areas) on a logarithmic scale. An interactive version of this figure with rates on a logarithmic and linear scale is available at https://kiana-k-collins.shinyapps.io/bmj_shiny_app_v2/. PSA values were categorised as above the age specific threshold if patients were aged 18-49 years with a PSA value >2.5 ng/mL, 50-59 years with a PSA value >3.5 ng/mL, 60-69 years with a PSA value >4.5 ng/mL, and ≥70 with a PSA value >6.5 ng/mL

The age standardised rate of PSA testing increased more than fivefold, from 11.8 (95% CI 11.7 to 12.0) in 2000 to 69.7 (69.5 to 70.0) in 2018. Rates slightly declined between 2014 and 2017. This trend was broadly similar across all sociodemographic characteristics (fig 2).

Over time, testing rates were consistently higher in patients aged 70-89 years. By year, testing rates for patients aged 70-79 years were three times higher than those aged 50-59 years (fig 2). The rate of testing was highest in patients of white ethnicity (55.9, 95% CI 55.9 to 56.0). Testing rates were highest in the South East of England (58.3, 58.2 to 58.4) and lowest in the North East (31.3, 31.1 to 31.5). Testing rates were more than twice as high in the least deprived groups compared with the most deprived groups (fig 2).

A 30% greater increase occurred in PSA values below the age specific thresholds (from 9.30 to 57.83 per 1000 person years) compared with those above the threshold (from 2.53 to 11.89 per 1000 person years). In 2018, the absolute rate of PSA testing for values below the referral threshold was 4.9 times higher than for values above it. Rates for PSA testing in patients with a family history of prostate cancer were 3.6 times higher than for those without a family history in 2000 and increased to 4.8 times by 2018. Testing rates overall were two to three times higher in asymptomatic patients (35.1, 35.2 to 35.2 per 1000 person years) than in patients with symptoms (12.9, 12.9 to 12.9 per 1000 person years). From 2017 and 2018 the standardised rate of PSA testing increased by 26.4% in asymptomatic patients and by 19.6% in patients with symptoms recorded (fig 2 and supplementary file 2).

### PSA testing rates by individual characteristics

Characteristics statistically significantly (P<0.001) associated with the likelihood of undergoing PSA testing were age, ethnicity, region, deprivation, family history of prostate cancer, ever having a PSA value above the age specific threshold, and ever having a symptom before a PSA test (table 2).

**Table 2:** Rate ratios of PSA testing: univariate and multivariable mixed effects negative binomial regression models

	Univariate models			Multivariable models	
	Rate ratio (95% CI)	P value		Rate ratio (95% CI)	P value
**Region (reference south east)**					
Intercept (rate)*	0.05 (0.04 to 0.05)			0.07 (0.06 to 0.07)	
East Midlands	0.78 (0.64 to 0.96)	<0.001		0.85 (0.73 to 0.99)	<0.001
East of England	1.00 (0.82 to 1.22)			0.89 (0.77 to 1.03)	
London	0.60 (0.54 to 0.67)			0.90 (0.83 to 0.97)	
North East	0.57 (0.48 to 0.69)			0.63 (0.55 to 0.72)	
North West	0.77 (0.70 to 0.86)			0.86 (0.80 to 0.93)	
South West	0.95 (0.83 to 1.08)			0.88 (0.80 to 0.97)	
West Midlands	0.83 (0.74 to 0.92)			0.91 (0.84 to 0.98)	
Yorkshire and the Humber	0.67 (0.55 to 0.81)			0.74 (0.64 to 0.86)	
Unknown	0.28 (0.12 to 0.66)			0.39 (0.21 to 0.74)	
**Age range (reference 60-69 years)**					
Intercept (rate)*	0.12 (0.12 to 0.13)			-	
18-29	0.01 (0.01 to 0.01)	<0.001		0.04 (0.03 to 0.04)	<0.001
30-39	0.09 (0.09 to 0.09)			0.18 (0.18 to 0.18)	
40-49	0.30 (0.30 to 0.30)			0.44 (0.44 to 0.44)	
50-59	0.67 (0.67 to 0.67)			0.77 (0.76 to 0.77)	
70-79	0.99 (0.98 to 0.99)			1.02 (1.02 to 1.03)	
80-89	0.77 (0.76 to 0.77)			1.01 (1.01 to 1.02)	
≥90	0.47 (0.45 to 0.48)			0.89 (0.86 to 0.91)	
**Ethnicity (reference white patients)**					
Intercept (rate)*	0.04 (0.04 to 0.04)			-	
Asian	0.64 (0.63 to 0.65)	<0.001		1.03 (1.01 to 1.04)	<0.001
Black	1.18 (1.17 to 1.19)			1.39 (1.37 to 1.40)	
Mixed	0.59 (0.58 to 0.60)			1.08 (1.06 to 1.10)	
Other	0.71 (0.70 to 0.72)			1.12 (1.11 to 1.14)	
South Asian	0.79 (0.78 to 0.80)			1.16 (1.15 to 1.17)	
Unknown	0.27 (0.27 to 0.27)			0.47 (0.47 to 0.48)	
**Index of multiple deprivation (reference category 1 (least deprived))**					
Intercept (rate)*	0.05 (0.05 to 0.05)			-	
2	0.88 (0.88 to 0.89)	<0.001		0.93 (0.92 to 0.93)	<0.001
3	0.78 (0.78 to 0.79)			0.87 (0.87 to 0.88)	
4	0.70 (0.70 to 0.71)			0.82 (0.82 to 0.82)	
5	0.62 (0.61 to 0.62)			0.75 (0.74 to 0.75)	
Unknown	0.57 (0.54 to 0.61)			0.78 (0.74 to 0.82)	
**PSA value ever above age specific threshold (reference no)†**					
Intercept (rate)*	0.03 (0.03 to 0.03)			-	
Yes	12.53 (12.44 to 12.61)	<0.001		3.84 (3.82 to 3.85)	<0.001
**Family history of prostate cancer (reference no)**					
Intercept (rate)*	0.03 (0.03 to 0.04)			-	
Yes	4.11 (4.01 to 4.21)	<0.001		3.24 (3.19 to 3.29)	<0.001
**Symptom ever present before a PSA test (reference no)**					
Intercept (rate)*	0.02 (0.02 to 0.02)			-	
Yes	12.50 (12.45 to 12.55)	<0.001		4.92 (4.90 to 4.94)	<0.001

PSA=prostate specific antigen.

Rate ratios were estimated from a mixed effects negative binomial regression model with a log-link function. The model included a random intercept for general practice and was adjusted for all covariates listed.

* Multivariable model intercept shows baseline rate for reference categories: all other values are rate ratios versus this baseline. Univariate model intercepts examine each factor separately.

† Above the threshold if aged 18-49 years with a PSA value >2.5 ng/mL, aged 50-59 years with a PSA value >3.5 ng/mL, aged 60-69 years with a PSA value >4.5 ng/mL, and aged ≥70 years with a PSA value >6.5 ng/mL.

Patients who were of an ethnicity other than white were more likely than those of white ethnicity to undergo PSA testing, with rate ratios ranging from 1.03 (95% CI 1.01 to 1.04) for patients of Asian ethnicity to 1.39 (1.37 to 1.40) for patients of black ethnicity (P<0.001) (table 2). Patients in the North East of England were less likely than patients in the South East to have a PSA test (0.63, 0.55 to 0.72; P<0.001). Patients in the most deprived areas of England were tested less frequently than patients in the least deprived areas (0.75, 0.74 to 0.75) (table 2). Having ever had a PSA value above the age specific threshold was associated with an increased rate ratio for PSA testing of 3.84 (3.82 to 3.85). Family history of prostate cancer was associated with an increased rate ratio for PSA testing of 3.24 (3.19 to 3.29).

The strongest association was for ever having a symptom recorded before a PSA test (4.92, 4.90 to 4.94; table 2). Overall, 45.8% (696 103) of patients with at least one PSA test also had at least one symptom recorded in the 90 days before one of their tests and 26.8% (1 029 253) of all PSA tests were paired with a symptom. Of these tests, 56.6% (582 652) were paired with lower urinary tract symptoms, 14.5% (149 274) with back pain, and 10.1% (103 873) with haematuria. Of the 785 366 patients who only had one test during the study period, 34.8% (273 442) of the tests were paired with a symptom. Of the 735 750 patients with multiple PSA tests, 24.2% (177 815) of the PSA tests were paired with a symptom.

### PSA retesting intervals

Of the 735 750 patients with at least two PSA tests, 3 050 074 PSA tests provided 2 314 324 retesting intervals. The median retesting interval before adjustment was 12.6 (IQR 6.2-27.5) months (see supplementary file 1). Once adjusted, the estimated geometric mean interval between tests was 19.3 months (table 3).

**Table 3:** Linear mixed effect models for length of PSA retesting intervals: multivariable models*

	Multivariable model overall				Multivariable model subgroup analysis		
	Interval ratios (95% CI)	Expected interval (months)*	P value		Interval ratios (95% CI)	Expected interval (months)*	P value
Intercept†	19.34 (18.92 to 19.69)		<0.001		21.60 (21.17 to 22.03)		<0.001
Region (reference south east):							
East Midlands	0.98 (0.93 to 1.03)	18.89	<0.001		1.01 (0.96 to 1.07)	21.81	<0.001
East of England	1.03 (0.99 to 1.08)	19.96			1.03 (0.98 to 1.08)	22.18	
London	0.98 (0.96 to 1.01)	19.04			0.94 (0.92 to 0.97)	20.37	
North East	1.05 (1.00 to 1.10)	20.25			1.06 (1.01 to 1.11)	22.82	
North West	0.95 (0.93 to 0.98)	18.41			0.97 (0.95 to 1.00)	21	
South West	1.02 (0.99 to 1.05)	18.89			1.06 (1.03 to 1.09)	22.89	
West Midlands	1.05 (1.02 to 1.07)	20.22			1.03 (1.00 to 1.06)	22.28	
Yorkshire and the Humber	0.97 (0.93 to 1.02)	18.85			0.99 (0.94 to 1.04)	21.36	
Unknown	0.93 (0.72 to 1.20)	17.93			0.90 (0.67 to 1.21)	19.41	
Age range (reference 60-69 years):							
18-29	0.80 (0.75 to 0.85)	15.39	<0.001		0.78 (0.73 to 0.83)	16.78	<0.001
30-39	1.42 (1.39 to 1.45)	27.49			1.40 (1.37 to 1.43)	30.21	
40-49	1.53 (1.52 to 1.54)	29.54			1.49 (1.48 to 1.50)	32.14	
50-59	1.26 (1.26 to 1.27)	24.44			1.25 (1.25 to 1.26)	27.1	
70-79	0.77 (0.76 to 0.77)	14.83			0.77 (0.77 to 0.77)	16.64	
80-89	0.68 (0.68 to 0.68)	13.16			0.62 (0.61 to 0.62)	13.29	
≥90	0.57 (0.56 to 0.58)	10.95			0.44 (0.43 to 0.46)	9.59	
Ethnicity (reference white patients):							
Asian	0.88 (0.87 to 0.90)	17.10	<0.001		0.86 (0.85 to 0.88)	18.61	<0.001
Black	0.89 (0.88 to 0.90)	17.27			0.87 (0.86 to 0.88)	18.77	
Mixed	0.88 (0.85 to 0.90)	17.00			0.87 (0.84 to 0.89)	18.72	
Other	0.90 (0.88 to 0.91)	17.37			0.88 (0.86 to 0.89)	18.9	
South Asian	0.91 (0.90 to 0.92)	17.55			0.89 (0.87 to 0.90)	19.14	
Unknown	0.87 (0.86 to 0.88)	16.83			0.82 (0.81 to 0.83)	17.77	
Index of multiple deprivation (reference category 1 (least deprived)):							
2	1.00 (0.99 to 1.00)	19.28	<0.001		0.99 (0.99 to 1.00)	21.42	<0.001
3	0.99 (0.99 to 1.00)	19.19			0.98 (0.97 to 0.99)	21.18	
4	0.98 (0.97 to 0.99)	18.97			0.97 (0.96 to 0.98)	20.88	
5	0.97 (0.96 to 0.98)	18.75			0.95 (0.94 to 0.95)	20.41	
Unknown	1.02 (0.96 to 1.09)	19.71			1.02 (0.94 to 1.09)	21.92	
PSA value ever above age specific threshold (reference no)‡	0.32 (0.32 to 0.33)	6.27	<0.001				<0.001
Family history of prostate cancer (reference no)	0.84 (0.82 to 0.85)	16.18	<0.001		0.83 (0.82 to 0.85)	18.03	<0.001
Presenting symptoms (reference no):							
Fatigue	0.99 (0.98 to 1.00)	19.17	0.13		0.99 (0.98 to 1.00)	21.4	0.16
Bone pain	0.97 (0.92 to 1.02)	18.74	0.23		0.96 (0.91 to 1.02)	20.8	0.22
Back pain	1.03 (1.02 to 1.04)	19.92	<0.001		1.04 (1.03 to 1.04)	22.35	<0.001
Unexpected weight loss	0.96 (0.94 to 0.98)	18.51	<0.001		0.94 (0.92 to 0.96)	20.38	<0.001
Haematuria	0.94 (0.93 to 0.95)	18.18	<0.001		0.96 (0.95 to 0.97)	20.72	<0.001
Erectile dysfunction	1.04 (1.03 to 1.05)	20.12	<0.001		1.04 (1.03 to 1.05)	22.44	<0.001
Lower urinary tract symptoms	1.02 (1.02 to 1.03)	19.76	<0.001		1.04 (1.04 to 1.05)	22.52	<0.001

CI=confidence interval; PSA=prostate specific antigen.

Months refers to the estimated geometric mean time between repeat PSA tests. For each covariate level, values were calculated by multiplying the reference interval by the exponentiated fixed effect, giving the estimated interval in months while holding all other covariates at their reference level.

* Univariate models are provided in supplementary file 1.

† The intercept represents the estimated geometric mean interval in months between PSA tests for the reference category (age 60-69 years, white patient, first fifth of index of multiple deprivation (least deprived), south E=east, no family history, no symptoms, never above PSA age specific threshold). In the overall model this was 19.34 months (95% CI 18.92 to 19.69) and in the subgroup model 21.60 months (95% CI 21.17 to 22.03).

‡ Above the threshold included if aged 18-49 years with a PSA value >2.5 ng/mL, aged 50-59 years with a PSA value >3.5 ng/mL, aged 60-69 years with a PSA value >4.5 ng/mL, and aged ≥70 years with a PSA value >6.5 ng/mL.

Patients aged 40-49 years had the longest estimated mean interval, at 29.5 months, and the interval decreased with age from 40 years (table 3). All ethnic groups had shorter PSA retesting intervals compared with patients of white ethnicity, ranging from 1.79 months shorter for patients of South Asian ethnicity (17.55 *v* 19.34 months) to 2.34 months shorter for those of mixed ethnicity (17.00 *v* 19.34 months). Regional variation was modest, with only one month longer or shorter intervals across regions. Compared with the South East, the North East had the longest retesting interval, at 0.91 months longer (20.25 *v* 19.34 months). Deprivation showed minimal variation, with a statistically significant but small difference of less than a month between the five groups (table 3).

The strongest predictor of a shorter retesting interval was a PSA value above the age specific threshold, which reduced the retesting interval by more than 13 months. A family history of prostate cancer was associated with a reduced retesting interval by three months (3.16 months) (table 3).

PSA tests paired with erectile dysfunction, back pain, and lower urinary tract symptoms had statistically significantly longer PSA retesting intervals, whereas PSA tests paired with unexpected weight loss and haematuria had statistically significantly shorter intervals. Haematuria showed the largest effect and was associated with a reduced PSA retesting interval by 1.16 months (table 3).

### Subgroup analysis

Seventy three per cent (535 990/735 750) of patients with multiple PSA tests never presented with a PSA value above the age specific threshold. These patients were included in the sub analysis and contributed 7 899 475 person years of observation, with a median follow-up of 17.2 (IQR 11.1-19.0) years (table 1). In total, 1 887 390 tests were performed, providing 1 351 400 retesting intervals. The median interval between tests was 17.8 (IQR 10.8-36.2) months (supplementary file 1). Similar trends for ethnicity in the main retesting intervals analysis were found in the sub analysis, but with a greater effect (table 3). Regional variation increased, with intervals ranging from 1.22 months longer in the North East compared with South East (22.82 *v* 21.60 months), to 1.23 months shorter in London (20.37 *v* 21.60 months) (table 3).

## Discussion

Between 2000 and 2018, of the 10 235 805 male patients in primary care in England older than 18 years, 14.9% (1 521 116) had at least one PSA test, increasing to 33.3% (1 313 394) for those older than 50 years (3 938 402). Half of these patients (735 750/1 521 116) had multiple PSA tests and 72.8% (535 990/735 750) never presented with a PSA value above the age specific threshold.^2^ The median retesting interval overall was 12.6 (IQR 6.2-27.5) months. Testing rates varied by region, deprivation, ethnicity, family history, age, PSA value above the age specific threshold, and whether patients ever had a symptom before any PSA test (P<0.001). Once tested, patients had shorter retesting intervals if they were older, were of an ethnicity other than white, had a family history of prostate cancer, or had previously raised PSA levels (P<0.001). Despite considerable variation in testing rates by region and deprivation, the length of retesting intervals was similar across these groups.

### Strengths and limitations of this study

Using data from 1.5 million patients over a follow up period of 19 years, this study comprehensively analysed PSA testing and length of retesting intervals in English primary care. Unlike earlier studies that focused on first PSA tests, we used mixed effects modelling to account for clustering by practice and examined repeat testing. Data linkages with HES, ONS, and NCRAS enabled us to investigate data from across the healthcare network. This study also provided new insights into the factors and symptoms linked to PSA testing and length of retesting intervals.

Studies using routinely collected primary care data have limitations, as analyses depend on what clinicians choose to record and code. Data on symptom presentation can be recorded in the free text of primary care records but are not accessible for research in CPRD at present. Both coded and free text data are available in the National Prostate Cancer Audit, which showed that only 19% of prostate cancer diagnoses were for asymptomatic patients.^17^ Without access to free text, we inferred the general practitioner’s (GP) reasons for PSA testing from coded symptoms.

Analyses of retesting intervals were limited to patients with at least two PSA tests during follow-up. This raises the possibility of censoring bias, as the sample is biased towards individuals who were already engaged in regular testing. Owing to constraints during follow-up, the estimated retesting intervals may be shorter than the true intervals for the broader population. To assess this, we ran two sensitivity analyses restricted to patients with at least six years of follow-up (the median). The first analysis defined follow-up from cohort entry. The estimated interval was 19.3 months in the full cohort, compared with 19.8 months. The direction and magnitude of associations remained consistent across models, suggesting the main findings are robust, but they reflect testing patterns among patients already undergoing repeat PSA testing, rather than the full population of patients undergoing PSA testing.

We did not include PSA tests that occurred before the patient was registered with the current general practice as we did not know where the patient was registered before joining the contributing CPRD general practice. As a result, we excluded 157 938 (3%) PSA tests that were recorded between 2000 and 2018. As this proportion is quite small, we do not expect it to have an impact on the findings and conclusions. Additionally, in the negative binomial model we adjusted for ever having a raised PSA value, which lies on the causal pathway to increased PSA testing. This may reduce observed associations with patient factors. Given that raised PSA levels appropriately trigger repeat testing, including this variable helps capture the real world patterns of PSA monitoring.

### Comparison with other studies

We report similar population based PSA testing rates over time as those previously reported.^14^ ^15^ ^18^ PSA testing increased rapidly in the early 2000s, followed by a plateau and decline in the mid to late 2010s. The US Preventive Services Task Force changed its recommendation in 2012 from encouraging PSA testing to advising against it.^19^ These trends may reflect shifts in English clinical practice in response to changes in international guidance on PSA testing, despite no material change in English guidelines. In France between 2006 and 2018, it was found that 50% of men had received five PSA tests, with the first test between the ages of 65 and 75 years. High PSA test use has been found in Canada,^20^ France,^21^ Ireland,^22^ and Sweden, where 75% of men aged 60 to 70 years had a PSA test in the preceding decade.^23^ The PSA testing rate in Australia increased by 9% annually between 2002 and 2007 and then decreased by 5% annually from 2007 to 2018.^24^

Our results are consistent with the findings from other longitudinal primary care studies conducted in the UK, which identified similar associations between PSA testing and age, ethnicity, level of deprivation, family history, and region.^13^ ^14^ ^15^ ^25^ ^26^ We report that patients of black ethnicity were more likely to have a PSA test than patients of white ethnicity and had shorter PSA retesting intervals (2.07 months) (table 3). In another large English primary care database (QResearch), it was found that rates of opportunistic PSA screening were statistically significantly associated with black ethnicity.^15^ We found variation in PSA testing rates at practice level, which was smaller but comparable to the 13% variation in PSA testing of asymptomatic patients found using Prostate Cancer Audit data.^17^ We observed less practice variation when examining the length of retesting intervals.

We found 26.8% (1 029 253) of all PSA tests (3 835 440) were paired with a symptom. A study assessing opportunistic PSA screening in the UK, identified that 65% of all first PSA tests in the study period were deemed to be for screening.^15^ We know of no other studies focusing on symptoms in primary care records before a PSA test.

### Policy implications

In early 2018, British media personalities Bill Turnbull and Stephen Fry publicly announced their prostate cancer diagnoses and shared their PSA testing and treatment journeys. From 2017 and 2018, PSA testing rates increased substantially: testing increased by 26.4% for asymptomatic patients and 19.6% for patients with symptoms, contrasting with declines of more than 4% for both these patient populations in the previous year. Similar patterns have been observed internationally. For example, Angelina Jolie’s disclosure that she has the *BRCA1 *gene led to increased genetic testing.^27^ While it is unclear if increased PSA testing led to more clinically significant prostate cancer diagnoses, one study reported a 30% increase in two week wait referrals and more diagnostic multiparametric MRIs (mpMRIs) and biopsies.^28^ These findings suggest that public health-seeking behaviours may shift during periods of high profile media attention, and health systems should anticipate for potential unpredictable surges in PSA testing, overtesting, and associated costs.

Most international guidelines recommend retesting asymptomatic patients aged 50 years every two to four years if the PSA value is between 1 and 3 ng/mL, and extending the retesting interval to 4-10 years if PSA is less than 1 ng/mL.^29^ ^30^ ^31^ ^32^ ^33^ ^34^ ^35^ ^36^ ^37^ These recommendations are based on indirect evidence from studies investigating a single PSA value, assessments of risk of prostate cancer progression, or data from randomised screening trials primarily aimed at mortality reduction, not retesting intervals.^10^ Australia’s 2025 draft guidelines strongly recommend that GPs offer PSA testing every two years for patients aged 50-69 years. The lack of a consistent international approach to PSA retesting underscores the need for evidence based recommendations reflect patient risk and the primary care setting.

### Research implications

We report considerable variation in PSA testing rates but less variation in the length of PSA retesting intervals. Although many patients were never tested, suggesting possible undertesting, others were tested only once, which may be insufficient for those at risk. We also identified a cohort of patients with low PSA values who underwent frequent testing beyond guideline recommendations, raising concerns about overtesting. The benefit of retesting and ad hoc screening remains uncertain and requires further research to determine evidence based retesting intervals that balance the benefits of early detection with the harms of overdiagnosis.

Unlike structured bowel or breast cancer screening, guidance on PSA retesting for patients with low PSA values is inconsistent. This raises concerns about equity and the psychological impacts of PSA testing for patients and their families, possibly leading to confusion and mistrust, with patients left to seek testing or interpret uncertain advice. Shared decision making promotes a patient centred approach to navigate the trade-offs between the benefits and harms of PSA testing. With shared decision making, the choice to undergo PSA testing is often patient driven, and factors such as education level and health literacy considerably influence these choices.^38^ ^39^ It is important that all patients understand the harms and benefits to make their own decisions on whether to engage with testing. Sweden has organised approaches to PSA testing, and patients still report a need for clearer communication and support.^40^ Research is required to understand patients’ and practitioners’ perspectives on PSA testing and retesting to develop strategies for shared decision making to mitigate the impact of unclear clinical consensus on the use of PSA tests.

Community based PSA testing is becoming more common in England. These are often through mobile PSA testing clinics run by charities such as CHAPS, a prostate cancer charity that runs three to six PSA testing events a month in different locations,^41^ and The Prostate Project’s “Man Van” initiative.^42^ As these PSA tests are delivered outside of NHS primary care, we may have underestimated how frequently patients have PSA tests in the community. If patients have a high PSA value at a separate clinic, we expect they would consult a GP and have a repeat test, but if not, this could mean many patients have already had PSA tests or we are missing those with low values who do not present to their GP. Future research should link PSA testing data from community based initiatives with primary care and hospital records to ensure complete capture of PSA testing episodes, enabling more accurate assessments of testing frequency, follow-up patterns, and subsequent diagnostic or treatment pathways.

No guidance for PSA retesting intervals or when to stop testing exists in England. Testing is typically offered to patients who present with symptoms or request a PSA test. More than 70% of 735 750 patients with multiple PSA tests never exceeded the age specific threshold. The subgroup analysis was done on this cohort of patients. Their median unadjusted retesting interval was 17.6 months (IQR 10.6-36.1). This interval is shorter than guideline recommended frequencies of two to four years for higher risk patients and represents frequent testing for lower risk patients who could be safely tested at much longer intervals. Research is needed to determine when it is safe to stop PSA testing considering age and PSA history. While some countries have established guidance on discontinuing PSA testing at age 70 years,^29^ ^30^ ^36^ ^43^ ^44^ or based on life expectancy,^29^ ^31^ ^32^ ^35^ ^37^ ^44^ these recommendations rely mainly on European screening trials^45^ ^46^ or American cohorts,^47^ highlighting the need either for evidence from primary care data in England or for guideline developers to consider this evidence as sufficient.

### Clinical implications

We found that PSA testing is closely aligned with changes in prostate cancer incidence. PSA testing rates in England declined sharply in 2015, with an annual percentage change of −7%, mirroring a similar drop in prostate cancer incidence.^48^ Declines in PSA testing may either reduce overdiagnosis and overtreatment or conversely risk delayed identification of clinically significant prostate cancers.

We found a cohort of younger patients outside the typical eligibility criteria for screening trials who were tested for PSA level. There were 63 903 PSA tests performed in patients under 40 years old, involving 56 914 individuals. Most of these patients (82.6% 47 010) had a single test. Of the tests in this age group, 42.8% (27 333) were paired with symptoms, most commonly lower urinary tract symptoms, erectile dysfunction, and back pain. With home based PSA testing kits now available through private providers, we anticipate increased testing in this age range. This represents an important cohort of patients who may experience harms of overtesting.

PSA testing rates were three times higher in patients in their 70s and 80s compared with those in their 50s, with retesting intervals up to a year shorter, even after accounting for PSA levels, symptoms, and personal characteristics. Patients older than 70 years are the least likely to benefit from repeat testing: they account for an estimated 40% of overdiagnoses,^49^ and many countries advise against testing this group.^29^ ^30^ ^36^ ^43^ ^44^ Some of the increased testing in older patients may reflect symptom presentation, which is consistent with guidance. Despite UK recommendations to limit PSA testing to patients with symptoms, or after discussion with a GP, we still observed considerable testing in asymptomatic patients. Current practice may not effectively target testing to those most likely to benefit, raising concerns about overdiagnosis.

### Conclusions

PSA testing in primary care is varied. Among patients who underwent multiple tests, many were tested more frequently than recommended, raising concerns about overtesting. PSA retesting is occurring in patients without recorded symptoms or with previous low PSA values. To ensure maximum patient benefit while reducing the risk of overtesting, research is urgently needed to determine appropriate evidence based PSA retesting intervals.

**What is already known on this topic**

The utility of repeat prostate specific antigen (PSA) testing for asymptomatic patients is unknown
Guidance for PSA retesting intervals before a diagnosis of prostate cancer varies internationally
PSA testing rates across England show regional variation, with lower testing rates observed in areas of higher deprivation

**What this study adds**

In this study of PSA testing in primary care in England, one quarter of all tests were paired with a symptom of prostate cancer
Of patients with multiple PSA tests, >70% never presented with a raised PSA value
More than two thirds of PSA tests were repeated within two and a half years

## Data Availability

This study is based on CPRD data and is subject to a full licence agreement, which does not permit data sharing outside of the research team. Code lists are available in supplementary file 4. The R code used for analysis is available on GitHub: https://github.com/kiana-k-collins/PSA-testing-and-retesting-trends-in-primary-care-in-England-between-2000-and-2018.

## Web extra

Extra material supplied by authors

Web appendix 1: Additional file 1

Web appendix 2: Additional file 2

Web appendix 3: Additional file 3

Web appendix 4: Additional file 4

## Notes

Contributors: RP and BDN contributed equally. KKC conceived and oversaw the study and developed the code lists for use in the study with guidance from BDN. KKC conducted data management and statistical analysis with guidance from JLO. KKC interpreted the statistical analysis with guidance from BDN and RP. PSV conducted data management for the BLOod Test Trend for cancEr Detection (BLOTTED) study. BDN and RP supervised this study. KKC wrote the first draft of the manuscript. All members of the team helped draft and commented on further revisions of the manuscript. All authors read and approved the final manuscript. KKC is the guarantor. Artificial intelligence in the form of ChatGPT was used to ensure that R used computing power as efficiently as possible. The corresponding author attests that all listed authors meet authorship criteria and that no others meeting the criteria have been omitted. The corresponding author attests that all listed authors meet authorship criteria and that no others meeting the criteria have been omitted.

Transparency: The lead author (KKC) affirms that the manuscript is an honest, accurate, and transparent account of the study being reported, that no important aspects of the study have been omitted, and that any discrepancies from the study as planned (and, if relevant, registered) have been explained.

Dissemination to participants and related patient and public communities: The results of this study will be disseminated through the media channels of the patient and public advisory group, the host institution of the lead author, and the funder for scientific and lay audiences. A lay summary is included in supplementary file 1.

Provenance and peer review: Not commissioned; externally peer reviewed.
