# Supplementary material for: Prostate specific antigen retesting intervals and trends in England: population based cohort study
Source: BMJ. 2025 Oct 8;391:e083800. doi: 10.1136/bmj-2024-083800 (PMC12505835; doi:10.1136/bmj-2024-083800)
Supplement: Supplementary file 1 — Web appendix 1: Additional file 1 [file colk083800.ww1.pdf]

Additional File 1

Lay summary

Lay summary: The PSA test is a blood test that detects a stressed prostate. A raised PSA value may be due to the presence of a prostate cancer tumour but it could also be due to many other urological conditions. The PSA test is not recommended for screening patients for prostate cancer because it is not accurate enough. Guidelines recommend different PSA retesting intervals, so this research aimed to understand who is being tested with PSA in England and how often. It was found that many patients undergo multiple PSA tests, even when they do not have symptoms of prostate cancer and when their PSA test results do not indicate a high risk of prostate cancer. This is important because it may be a sign of overtesting patients which causes harms and does not use health resources efficiently. Factors such as where people live, their age, ethnicity, and socioeconomic status significantly influenced how often PSA testing occurred but made less of a difference when looking at how soon retesting happened. We recommend developing clearer evidence-based guidelines for PSA testing and retesting to ensure that all patients receive consistent and appropriate care.

Detailed Variable Methodology

PSA threshold definition

PSA valued were retrieved from the patients primary care record (CPRD Aurum) using SNOMED-CT codes. PSA values were categorised as above the NICE NG12 (1) threshold if age 18-49 and PSA value >2.5 ng/ml, age 50-59 and PSA value >3.5 ng/ml, age 60-69 and PSA value > 4.5 ng/ml and aged 70+ and PSA value over 6.5 ng/ml.

Symptom recording

Symptoms were retrieved from the patients primary care record (CPRD Aurum) using SNOMED-CT codes. Symptoms recorded in the 90 days before the PSA test were included. If multiple PSA tests were taken within the 90-day time window, or multiple symptoms were recorded in the 90-day window, the closest symptom to the PSA test was included. If two symptoms were recorded on the same day a random one was chosen.

Ethnicity recording

Ethnicity was primarily retrieved from the HES database. If the same patient had multiple ethnicities coded in HES, the most common ethnicity was used. If a patient had two ethnicities or an equal amount of two different ethnicities, then a random ethnicity between the two was chosen. There is no date associated with ethnicity codes in HES. If ethnicity was not recorded in HES, we used SNOMED-CT codes to identify ethnicity in CPRD and used the patient’s most recent record. The combined ethnicity from HES and CPRD has been shown to be comparable to the ethnicity distribution in the UK (2). Over 6.5 million patients had an ethnicity recorded in CPRD and over 6.4 million had a record in HES . Over 4.4 million had a record in both HES and CPRD.

Cancer diagnosis recording

Prostate cancer diagnosis was defined by ICD10 code C61 in NCRAS and HES and by SNOMED-CT code in CPRD. The earliest date of cancer diagnosis from NCRAS was used as the primary source and if not recorded then the earliest date of diagnosis was taken between HES and CPRD.

Date of death

Date of death was retrieved from the ONS as the primary source. Any additional deaths were classified by the earliest date of death between CPRD and HES(3).

Figure 1: Flow chart of participant selection

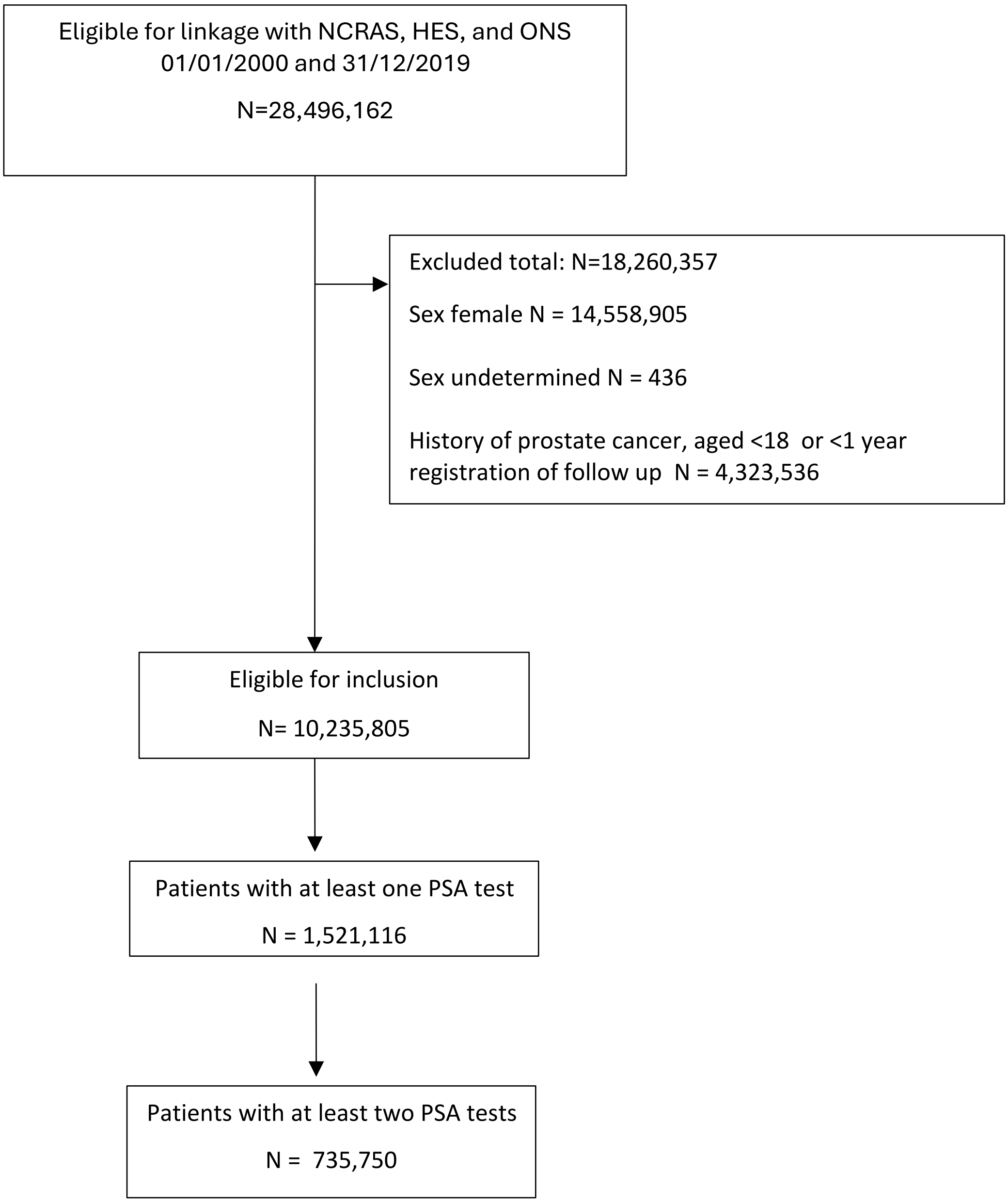

Table 2: Overall age-standardised rate of PSA tests per 1000 person years by year (N = 10,235,805)

Year	Crude Rate (per 1000 person years)	Standardised rate (per 1000 person years)	95% CI	% change from previous year
**2000**	11.10	11.83	11.71 -11.95	
**2001**	16.74	17.92	17.78 - 18.06	51%
**2002**	22.37	24.03	23.87 - 24.19	34%
**2003**	28.28	30.47	30.30 - 30.65	27%
**2004**	36.15	39.05	38.85 - 39.25	28%
**2005**	38.28	41.43	41.23 - 41.64	6%
**2006**	40.27	43.60	43.39 - 43.81	5%
**2007**	43.76	47.14	46.92 - 47.35	8%
**2008**	49.27	52.72	52.49 - 52.95	12%
**2009**	53.70	57.28	57.04 - 57.51	9%
**2010**	54.14	57.60	57.36 - 57.83	1%
**2011**	55.75	59.01	58.78 - 59.24	2%
**2012**	56.71	59.81	59.58 - 60.04	1%
**2013**	62.21	64.99	64.75 - 65.24	9%
**2014**	61.07	63.37	63.13 - 63.60	-3%
**2015**	57.33	59.17	58.94 - 59.39	-7%
**2016**	56.86	58.54	58.32 - 58.76	-1%
**2017**	54.51	55.95	55.73 - 56.16	-4%
**2018**	69.72	69.72	69.49 - 69.96	25%

Figure 2: Proportion of PSA tests with a symptom recorded in the 90 days before the test by symptom between 2000 and 2018

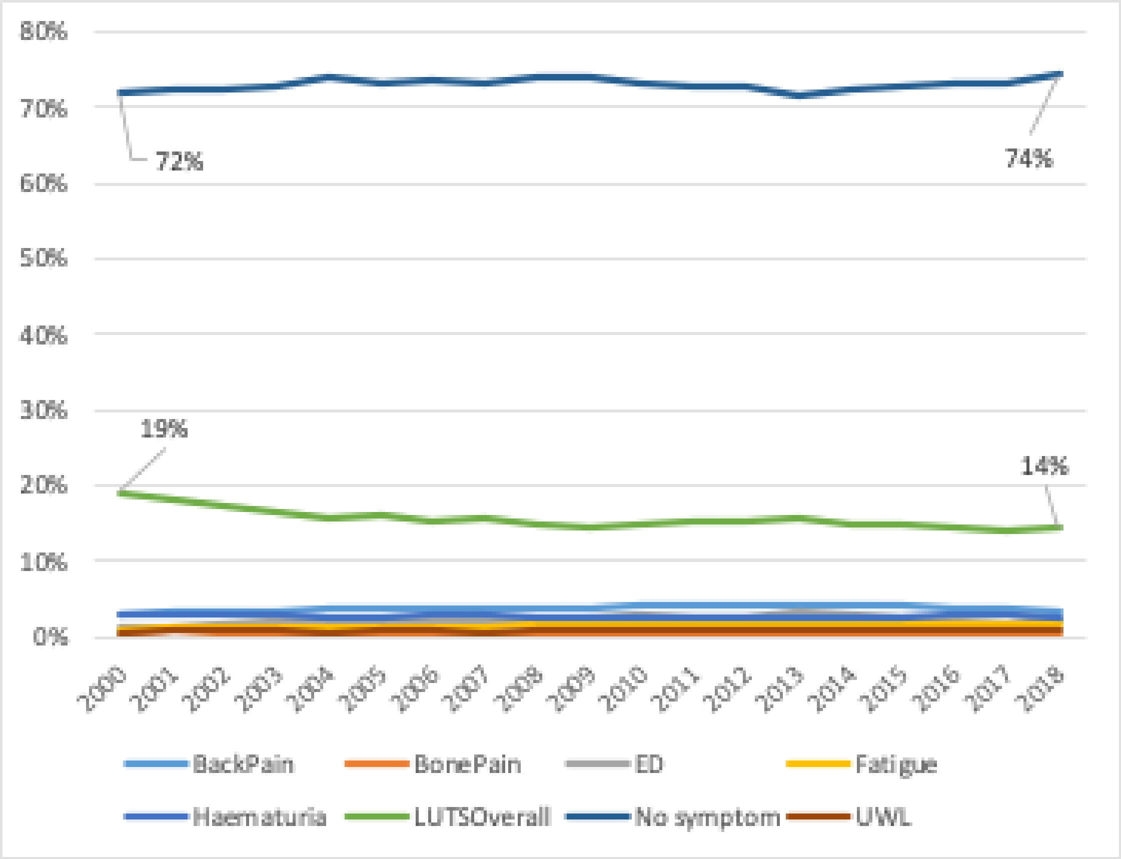

The proportion of PSA tests with symptoms recorded 0-90 days before the test remained stable throughout the 19 years of follow up, except LUTS declined from 19% - 14%.

Table 1a: Median intervals split by region, IMD, ethnicity, age, PSA value above/below the age specific referral threshold and symptoms

**Overall patients with at least two PSA tests (N = 735,750)**				
**Category**	**Number of distinct patients**	**Number of PSA retesting intervals**	**Median months until next test**	**Interquartile Range (IQR)**
**Region**				
East Midlands	18,725	61,601	12.12	5.98-25.85
East of England	37,425	117,456	13.07	6.47-28.38
London	115,415	325,729	13.86	7.46-28.32
North East	16,564	43,510	13.21	5.98-34.62
North West	131,290	412,233	12.52	6.08-27.17
South East	174,295	573,183	12.65	6.34-27.27
South West	91,470	309,641	11.86	5.75-25.76
West Midlands	126,852	398,842	12.84	6.41-27.99
Yorkshire and The Humber	23,575	71,841	12.61	6.18-28.32
**Age at PSA test**				

18-29	895	1,248	13.27	3.94-33.32
30-39	9,111	11,544	26.92	10.51-60.59
40-49	71,511	103,503	26.31	11.96-54.43
50-59	214,999	400,944	18.82	9.17-40.54
60-69	312,535	790,047	12.94	6.54-27.73
70-79	253,800	717,284	11.66	5.91-21.68
80-89	103,866	269,459	9.63	4.86-17.71
90+	10,147	20,295	7.26	3.61-14.29
**Ethnicity**				
Asian	8,848	25,037	13.57	7.36-27.37
Black	23,653	63,741	14.16	7.39-28.06
Mixed	3,447	9,446	13.73	6.93-29.07
Other	9,236	25,561	13.83	7.23-28.48
South Asian	21,586	61,127	13.76	7.49-28.29
Unknown	27,806	68,628	12.22	5.98-24.90
White	641,174	2,060,784	12.61	6.21-27.56
**Index of multiple deprivation (IMD)**				
1	193,930	671,011	12.48	6.24-26.61
2	173,569	575,797	12.45	6.21-26.77
3	146,258	458,767	12.65	6.21-27.33
4	124,324	355,329	13.04	6.31-28.58
5	96,972	251,373	13.63	6.47-30.26
Unknown	697	2,047	13.04	6.67-28.45
**Above age-specific threshold**				
Yes	172,752	540,270	5.75	2.69-10.81
No	655,159	1,774,054	15.60	9.23-33.11
**Family history**				
Yes	11,337	40,052	13.12	7.36 – 26.08
No	724,413	2,271,937	12.65	6.24 – 27.56
**Erectile dysfunction (ED)**				
No	725,123	2,265,450	12.65	6.24-27.30
Yes	41,645	48,874	16.89	7.88-37.12
**Haematuria**				
No	724,689	2,261,587	12.65	6.27-27.40
Yes	43,719	52,737	12.48	4.60-31.50
**Back Pain**				
No	720,165	2,234,883	12.61	6.24-27.27
Yes	66,964	79,441	15.47	7.26-34.10
**Bone Pain**				
No	735,523	2,312,725	12.65	6.24-27.53
Yes	1,556	1,599	12.65	6.24-27.46
**Lower urinary tract symptoms (LUTS)**				
No	662,726	1,987,002	12.42	6.21-26.25
Yes	220,397	327,322	15.93	6.96-34.95
**Unexpected weight loss (UWL)**				
No	732,712	2,301,846	12.65	6.24-27.50

Yes	11,410	12,478	14.49	6.37-32.29
**Fatigue**				
No	729,740	2,282,481	12.65	6.24-27.40
Yes	28,485	31,843	15.57	7.13-33.34

Caption: The overall median retesting interval across all categories was 12.6 months (IQR 6.2 to 27.5).

Table 1b: Median intervals split by region, IMD, ethnicity, age, PSA value above/below the age specific threshold and symptoms (subgroup analysis)

**Sub-analysis: Patients with at least two tests and never had a PSA result above the age-specific threshold and do not have prostate cancer (N = 535,990)**				
**Category**	**Number of distinct patients**	**Number of PSA retesting intervals**	**Median months until next test**	**Interquartile Range (IQR)**
**Region**				
East Midlands	13,501	34,545	16.92	9.92-36.14
East of England	26,933	68,094	18.53	11.07-36.89
London	87,594	212,460	17.28	10.55-33.61
North East	11,957	24,919	21.98	11.10-45.93
North West	96,860	246,367	17.28	10.51-35.58
South East	126,795	334,600	17.51	10.74-35.97
South West	63,080	157,051	18.56	10.91-38.37
West Midlands	92,260	232,247	18.17	11.04-36.63
Yorkshire and The Humbe r	16,917	40,973	18.13	11.04-38.11
**Age at PSA test**				
18-29	830	1,144	14.90	5.49-35.24
30-39	8,283	10,234	29.53	12.35-63.40
40-49	61,437	84,336	29.89	14.26-57.98
50-59	166,333	273,663	24.18	12.61-47.11
60-69	217,996	450,057	18.40	11.33-36.27
70-79	171,017	396,982	14.09	9.13-28.19
80-89	62,973	127,414	12.88	7.39-24.61
90+	4,926	7,570	11.73	6.04-20.37
**Ethnicity**				
Asian	7,225	18,065	16.10	9.66-31.47
Black	17,071	39,129	17.84	10.61-33.15
Mixed	2,617	5,988	18.05	10.68-35.53
Other	7,330	17,323	17.21	10.35-33.51
South Asian	18,180	46,143	15.90	9.43-31.80
Unknown	20,425	41,612	16.33	10.12-32.95
White	463,142	1,183,140	17.94	10.84-36.70
**Index of multiple deprivation (IMD)**				

1	139,142	380,109	17.58	11.01-35.94
2	125,612	331,422	17.44	10.78-35.81
3	106,207	265,805	17.77	10.74-36.14
4	91,717	213,606	18.17	10.58-36.56
5	72,795	159,266	18.43	10.35-37.02
Unknown	517	1,192	19.91	11.50-37.48
**Family history**				
Yes	8,430	25,206	16.49	11.27-31.77
No	527,560	1,326,194	17.81	10.74-36.23
**Erectile dysfunction (ED)**				
No	526,715	1,317,394	17.67	10.74-36.01
Yes	29,987	34,006	22.04	11.53-43.43
**Haematuria**				
No	528,115	1,323,893	17.71	10.78-36.07
Yes	24,274	27,507	20.60	9.72-43.10
**Back Pain**				
No	522,540	1,297,107	17.67	10.74-36.01
Yes	47,249	54,293	20.20	10.91-40.21
**Bone Pain**				
No	535,798	1,350,377	17.77	10.78-36.17
Yes	1,004	1,023	16.33	9.44-34.31
**Lower urinary tract symptoms (LUTS)**				
No	477,689	1,140,788	17.08	10.58-35.12
Yes	151,295	210,612	21.81	11.60-41.75
**Unexpected weight loss (UWL)**				
No	533,472	1,342,645	17.77	10.78-36.17
Yes	8,164	8,755	18.30	9.17-36.97
**Fatigue**				
No	530,823	1,329,646	17.74	10.78-36.14
Yes	19,867	21,754	20.01	10.61-38.86

Caption: The overall median retesting interval across all categories was 17.8 months (IQR 10.8 to 36.2).

Table 2: Linear mixed effect models for length of PSA retesting intervals: Univariate Models

	**Univariate model overall (all patients with at least two PSA tests N = 735,750)**				**Univariate sub-group analysis (N = 535,900)**			
	Interval ratios	95% CI	Expected	P value	**Exp(value)**	**95% CI**	**Expected**	**P value**

			months				**months**	
**Region (ref South East)**				<0.001				<0.001
Intercept (rate)*	15.14	14.48	15.47		20.29	19.89 – 20.71		
East Midlands	0.96	0.91 - 1.01	14.5		1.02	-0.04 - 0.07	20.68	
East of England	1.02	0.97 - 1.08	15.5		1.03	-0.02 - 0.08	20.88	
London	1.01	0.99 - 1.04	15.3		0.95	-0.08 - -0.03	19.26	
North East	1.04	0.99 - 1.09	15.7		1.06	0.02 - 0.11	21.50	
North West	0.96	0.93 - 0.98	14.5		0.98	-0.05 - 0.00	19.78	
South West	0.96	0.93 - 0.99	14.5		1.04	0.01 - 0.07	21.08	
West Midlands	1.03	1.01 - 1.06	15.6		1.02	-0.01 - 0.05	20.68	
Yorkshire and Humber	0.97	0.92 - 1.02	14.6		0.99	-0.06 - 0.04	20.06	
Unknown	0.67	0.51 - 0.89	10.2		0.74	-0.60 - 0.00	14.99	
**Age range (ref 60-69)**				<0.001				<0.001
Intercept*	15.34	15.24-15.45			20.72	20.51 – 20.93		
18-29	0.92	0.86 - 0.99	14.18		0.76	0.71 - 0.81	15.77	
30-39	1.65	1.61 - 1.69	25.36		1.37	1.35 - 1.40	28.49	
40-49	1.71	1.70 - 1.72	26.28		1.47	1.45 - 1.48	30.41	
50-59	1.35	1.34 - 1.35	20.72		1.25	1.24 - 1.25	25.83	
70-79	0.78	0.78 - 0.78	11.98		0.77	0.77 - 0.77	16	
80-89	0.59	0.59 - 0.60	9.14		0.61	0.61 - 0.62	12.75	
90+	0.42	0.41 - 0.43	6.47		0.44	0.43 - 0.45	9.15	
**Ethnicity (ref White)**				<0.001				<0.001
Intercept	15.14	15.00 - 15.28			20.51	20.33 – 2.70		
Asian	1.01	0.99 - 1.03	15.24		0.93	0.91 - 0.95	18.96	
Black	0.96	0.95 - 0.97	14.53		0.96	0.95 - 0.97	19.65	
Mixed	0.99	0.95 - 1.02	14.93		0.97	0.94 - 1.00	19.84	
Other	1.02	1.00 - 1.04	15.44		0.96	0.94 - 0.98	19.58	

South Asian	1.04	1.03 - 1.06	15.77		0.93	0.92 - 0.94	19.06	
Unknown	0.82	0.81 - 0.83	12.43		0.82	0.81 - 0.83	16.77	
**IMD (ref 1 least deprived)**				<0.001				<0.001
Intercept	15.31	15.15 – 15.47			20.51	20.31 – 20.71		
2	0.99	0.99 - 1.00	15.22		0.99	0.99 - 1.00	20.42	
3	0.99	0.98 - 0.99	15.09		0.98	0.98 - 0.99	20.25	
4	0.97	0.96 - 0.98	14.87		0.97	0.96 - 0.98	19.95	
5	0.96	0.95 - 0.97	14.7		0.95	0.95 - 0.96	19.64	
Unknown	1.02	0.95 - 1.11	15.66		1.04	0.96 - 1.12	21.33	
**PSA value above age-specific threshold (ref no)**				<0.001	-	-	-	-
Intercept	18.18	18.01 – 18.36			-	-	-	-
Yes	0.31	0.31 – 0.31	5.66		-	-	-	-
**Family history (ref no)**				<0.001				<0.001
Intercept	15.06	14.92 – 15.20			20.29	20.10 – 20.49		
Yes	1.06	1.04 – 1.06	15.99		1.00	0.98 – 1.01	20.09	
**Fatigue (reference no)**				0.28				0.09
**int**	15.07	14.93 – 15.21			20.29	20.10 – 20.49		
Yes	1.01	0.99 - 1.02	15.16		0.98	0.97 – 1.00	19.83	
**Bone Pain (ref no)**								0.01

**int**	15.07	14.93 – 15.21			20.29	20.10 – 20.49		
Yes	0.95	0.90 – 1.01	14.33		0.92	0.87 – 0.98	18.65	
**Back pain (ref no)**				<0.001				<0.001
**int**	15.02	14.89 – 15.16			20.09	19.90 – 20.28		
Yes	1.05	1.05 – 1.06	15.84		1.02	1.01 – 1.03	20.53	
**UWL (ref no)**				<0.001				<0.001
**int**	15.08	14.94 – 15.22						
Yes	0.92	0.90 – 0.94	13.80					
**Haematuria (ref no)**				<0.001				<0.001
**int**	15.14	15.00 – 15.28			20.23			
Yes	0.83	0.82 – 0.84	12.54		0.92	0.91 – 0.93	18.91	
**ED (ref no)**				<0.001				<0.001
**int**	15.00	14.87 – 15.14			20.09	19.90 – 20.28		
Yes	1.14	1.13 – 1.15	17.07		1.10	1.08 – 1.11	22.07	
**LUTS (ref no)**				<0.001				<0.001
**int**	15.01	14.88 – 15.15			20.09	19.90 – 20.28		
Yes	1.02	1.01 – 1.02	15.25		1.04	1.05 – 1.05	20.90	

1. National Institute for Health and Care Excellence (NICE) (2021) Suspected cancer recognition and referral NG12. Available from: https://www.nice.org.uk/guidance/ng12/chapter/Recommendations-organised-by-site-of-cancer#urological-cancers
2. Shiekh SI, Harley M, Ghosh RE, Ashworth M, Myles P, Booth HP, Axson EL. Completeness, agreement, and representativeness of ethnicity recording in the United Kingdom's Clinical Practice Research Datalink (CPRD) and linked Hospital Episode Statistics (HES). Population Health Metrics. 2023;21(1):3.
3. Gallagher AM, Dedman D, Padmanabhan S, Leufkens HGM, de Vries F. The accuracy of date of death recording in the Clinical Practice Research Datalink GOLD database in England

compared with the Office for National Statistics death registrations. Pharmacoepidemiol Drug Saf. 2019;28(5):563-9.
