# Supplementary material for: Prostate specific antigen retesting intervals and trends in England: population based cohort study
Source: BMJ. 2025 Oct 8;391:e083800. doi: 10.1136/bmj-2024-083800 (PMC12505835; doi:10.1136/bmj-2024-083800)
Supplement: Supplementary file 2 — Web appendix 2: Additional file 2 [file colk083800.ww2.pdf]

Additional File 2

Table 1: Temporal trends PSA age standardised testing rates by year and age per 1000 person-years between 2000 and 2018 (Data from Figure 2)

Age range	***Year***	***Standardised rate***	***Lower CI 95%***	***Upper CI 95%***
18-29	2000	0.09	0.07	0.11
30-39	2000	0.56	0.51	0.62
40-49	2000	3.30	3.17	3.45
50-59	2000	14.33	14.03	14.64
60-69	2000	30.33	29.80	30.85
70-79	2000	39.20	38.48	39.92
80-89	2000	38.70	37.49	39.94
90+	2000	25.81	23.03	28.84
18-29	2001	0.14	0.11	0.17
30-39	2001	0.93	0.86	0.99
40-49	2001	5.25	5.08	5.43
50-59	2001	21.20	20.84	21.57
60-69	2001	45.48	44.85	46.11
70-79	2001	60.08	59.21	60.95
80-89	2001	59.10	57.70	60.53
90+	2001	40.36	37.17	43.75
18-29	2002	0.17	0.14	0.20
30-39	2002	1.23	1.15	1.30
40-49	2002	6.84	6.65	7.03
50-59	2002	28.41	27.99	28.83
60-69	2002	61.61	60.88	62.34
70-79	2002	80.40	79.40	81.41
80-89	2002	78.61	77.04	80.22
90+	2002	55.58	51.91	59.44
18-29	2003	0.21	0.18	0.24
30-39	2003	1.59	1.51	1.68
40-49	2003	8.50	8.29	8.71
50-59	2003	34.97	34.51	35.43
60-69	2003	77.17	76.36	77.98
70-79	2003	104.36	103.22	105.50
80-89	2003	103.03	101.24	104.83
90+	2003	72.79	68.69	77.07
18-29	2004	0.33	0.29	0.37
30-39	2004	2.07	1.97	2.17
40-49	2004	10.61	10.38	10.84
50-59	2004	45.08	44.57	45.60
60-69	2004	99.64	98.73	100.55

70-79	2004	132.15	130.87	133.43
80-89	2004	131.51	129.52	133.53
90+	2004	100.97	96.19	105.92
18-29	2005	0.38	0.34	0.42
30-39	2005	2.33	2.23	2.44
40-49	2005	11.87	11.63	12.11
50-59	2005	47.66	47.13	48.19
60-69	2005	103.49	102.57	104.40
70-79	2005	141.07	139.76	142.40
80-89	2005	142.68	140.62	144.76
90+	2005	103.16	98.33	108.17
18-29	2006	0.41	0.36	0.45
30-39	2006	2.41	2.31	2.52
40-49	2006	12.66	12.42	12.91
50-59	2006	47.74	47.21	48.27
60-69	2006	109.03	108.10	109.96
70-79	2006	151.78	150.41	153.14
80-89	2006	151.46	149.37	153.57
90+	2006	117.56	112.45	122.85
18-29	2007	0.38	0.34	0.43
30-39	2007	2.63	2.53	2.74
40-49	2007	13.44	13.19	13.69
50-59	2007	51.24	50.70	51.79
60-69	2007	118.05	117.11	119.00
70-79	2007	164.59	163.18	166.01
80-89	2007	165.88	163.73	168.06
90+	2007	123.48	118.30	128.84
18-29	2008	0.52	0.48	0.57
30-39	2008	3.01	2.89	3.13
40-49	2008	15.86	15.59	16.12
50-59	2008	58.10	57.52	58.69
60-69	2008	131.31	130.33	132.29
70-79	2008	183.43	181.95	184.91
80-89	2008	181.17	178.95	183.42
90+	2008	135.02	129.57	140.64
18-29	2009	0.63	0.58	0.69
30-39	2009	3.72	3.59	3.85
40-49	2009	18.27	17.98	18.55
50-59	2009	65.23	64.62	65.84
60-69	2009	140.63	139.63	141.64
70-79	2009	197.10	195.57	198.63
80-89	2009	190.60	188.35	192.87
90+	2009	146.46	140.83	152.26

18-29	2010	0.65	0.60	0.71
30-39	2010	3.65	3.52	3.78
40-49	2010	18.96	18.67	19.24
50-59	2010	63.33	62.73	63.93
60-69	2010	141.59	140.59	142.59
70-79	2010	199.97	198.44	201.50
80-89	2010	194.43	192.18	196.70
90+	2010	153.68	148.17	159.34
18-29	2011	0.77	0.72	0.83
30-39	2011	3.99	3.86	4.13
40-49	2011	20.11	19.82	20.41
50-59	2011	64.67	64.07	65.27
60-69	2011	143.80	142.80	144.80
70-79	2011	202.37	200.84	203.92
80-89	2011	204.71	202.42	207.02
90+	2011	160.14	154.80	165.62
18-29	2012	0.82	0.76	0.88
30-39	2012	4.42	4.28	4.56
40-49	2012	20.60	20.30	20.90
50-59	2012	65.56	64.97	66.16
60-69	2012	143.32	142.33	144.32
70-79	2012	206.78	205.24	208.34
80-89	2012	209.30	207.01	211.61
90+	2012	157.90	152.78	163.15
18-29	2013	0.99	0.93	1.06
30-39	2013	4.74	4.59	4.89
40-49	2013	23.93	23.61	24.25
50-59	2013	71.91	71.30	72.53
60-69	2013	154.44	153.42	155.47
70-79	2013	225.15	223.55	226.75
80-89	2013	220.42	218.08	222.78
90+	2013	165.78	160.60	171.09
18-29	2014	0.99	0.93	1.05
30-39	2014	4.79	4.64	4.94
40-49	2014	23.42	23.10	23.74
50-59	2014	69.81	69.21	70.40
60-69	2014	147.76	146.77	148.76
70-79	2014	220.82	219.27	222.38
80-89	2014	221.35	219.03	223.69
90+	2014	162.74	157.70	167.90
18-29	2015	0.98	0.92	1.05
30-39	2015	4.58	4.44	4.72
40-49	2015	21.63	21.33	21.94

50-59	2015	63.43	62.88	63.99
60-69	2015	137.40	136.45	138.35
70-79	2015	207.48	206.00	208.97
80-89	2015	213.54	211.29	215.81
90+	2015	157.56	152.68	162.55
18-29	2016	0.98	0.92	1.05
30-39	2016	4.65	4.51	4.79
40-49	2016	21.52	21.21	21.82
50-59	2016	63.48	62.93	64.02
60-69	2016	132.82	131.89	133.75
70-79	2016	207.61	206.16	209.08
80-89	2016	211.32	209.11	213.55
90+	2016	158.79	153.99	163.70
18-29	2017	1.07	1.01	1.14
30-39	2017	4.73	4.59	4.87
40-49	2017	20.98	20.68	21.28
50-59	2017	60.77	60.24	61.30
60-69	2017	125.18	124.28	126.08
70-79	2017	198.38	196.99	199.77
80-89	2017	203.46	201.32	205.61
90+	2017	150.15	145.55	154.85
18-29	2018	1.07	1.01	1.14
30-39	2018	4.97	4.83	5.12
40-49	2018	25.17	24.84	25.51
50-59	2018	81.30	80.69	81.92
60-69	2018	161.55	160.53	162.58
70-79	2018	240.31	238.80	241.82
80-89	2018	238.41	236.10	240.74
90+	2018	164.23	159.43	169.14

Table 2: Temporal trends PSA age-standardised testing rates by year and ethnicity per 1000 person-years between 2000 and 2018 (Data from Figure 2)

Ethnicity	***Year***	***Standardised rate***	***Lower CI 95%***	***Upper CI 95%***
Asian	2000	5.44	4.64	6.62
Black	2000	7.86	7.06	8.83
Mixed	2000	3.87	2.92	5.94
Other	2000	7.85	6.91	8.95
South Asian	2000	5.58	5.04	6.18
Unknown	2000	5.45	5.29	5.61
White	2000	14.96	14.79	15.15
Asian	2001	7.69	6.81	8.75
Black	2001	12.86	11.82	14.02

Mixed	2001	8.20	6.82	10.34
Other	2001	12.09	10.97	13.35
South Asian	2001	8.28	7.70	8.92
Unknown	2001	8.16	7.97	8.36
White	2001	22.52	22.31	22.72
Asian	2002	11.41	10.37	12.58
Black	2002	16.00	14.89	17.22
Mixed	2002	10.80	9.30	12.67
Other	2002	13.83	12.69	15.06
South Asian	2002	11.81	11.13	12.54
Unknown	2002	10.11	9.89	10.33
White	2002	30.05	29.82	30.28
Asian	2003	13.38	12.33	14.54
Black	2003	19.07	17.93	20.30
Mixed	2003	13.02	11.44	14.85
Other	2003	16.27	15.09	17.53
South Asian	2003	14.91	14.17	15.68
Unknown	2003	12.59	12.34	12.85
White	2003	37.69	37.44	37.94
Asian	2004	18.90	17.69	20.20
Black	2004	27.36	26.09	28.70
Mixed	2004	17.84	16.06	19.83
Other	2004	23.27	21.90	24.70
South Asian	2004	21.54	20.69	22.44
Unknown	2004	15.29	15.00	15.58
White	2004	47.65	47.38	47.92
Asian	2005	21.84	20.60	23.14
Black	2005	33.05	31.74	34.43
Mixed	2005	18.96	17.21	20.88
Other	2005	25.84	24.45	27.29
South Asian	2005	25.93	25.03	26.87
Unknown	2005	15.41	15.11	15.72
White	2005	49.66	49.40	49.93
Asian	2006	24.18	22.95	25.48
Black	2006	36.59	35.29	37.94
Mixed	2006	21.63	19.84	23.55
Other	2006	27.49	26.11	28.92
South Asian	2006	28.62	27.70	29.58
Unknown	2006	15.20	14.88	15.52
White	2006	51.66	51.39	51.92
Asian	2007	25.73	24.51	27.00
Black	2007	40.66	39.34	42.02
Mixed	2007	23.45	21.66	25.36

Other	2007	28.80	27.45	30.21
South Asian	2007	31.97	31.03	32.94
Unknown	2007	14.89	14.55	15.24
White	2007	55.44	55.17	55.71
Asian	2008	28.44	27.22	29.71
Black	2008	47.29	45.93	48.68
Mixed	2008	25.61	23.83	27.49
Other	2008	32.15	30.78	33.58
South Asian	2008	36.83	35.84	37.84
Unknown	2008	15.06	14.70	15.43
White	2008	61.67	61.39	61.95
Asian	2009	32.78	31.52	34.09
Black	2009	53.55	52.15	54.97
Mixed	2009	30.62	28.74	32.61
Other	2009	36.65	35.23	38.11
South Asian	2009	41.73	40.71	42.78
Unknown	2009	15.97	15.58	16.37
White	2009	66.36	66.08	66.65
Asian	2010	32.69	31.48	33.94
Black	2010	55.83	54.46	57.24
Mixed	2010	30.54	28.72	32.45
Other	2010	37.51	36.13	38.92
South Asian	2010	42.29	41.29	43.31
Unknown	2010	15.54	15.14	15.95
White	2010	66.32	66.03	66.60
Asian	2011	33.25	32.08	34.45
Black	2011	57.41	56.07	58.79
Mixed	2011	32.01	30.22	33.89
Other	2011	37.84	36.50	39.22
South Asian	2011	43.36	42.37	44.36
Unknown	2011	16.67	16.24	17.10
White	2011	67.51	67.23	67.80
Asian	2012	33.38	32.26	34.54
Black	2012	59.06	57.74	60.40
Mixed	2012	31.46	29.74	33.25
Other	2012	38.16	36.86	39.49
South Asian	2012	43.12	42.17	44.09
Unknown	2012	16.92	16.48	17.37
White	2012	68.22	67.94	68.50
Asian	2013	33.84	32.75	34.96
Black	2013	63.67	62.35	65.00
Mixed	2013	32.33	30.65	34.08
Other	2013	40.74	39.45	42.07

South Asian	2013	45.47	44.52	46.44
Unknown	2013	18.90	18.42	19.38
White	2013	74.01	73.72	74.30
Asian	2014	34.19	33.13	35.28
Black	2014	65.25	63.97	66.55
Mixed	2014	32.49	30.88	34.17
Other	2014	41.59	40.33	42.88
South Asian	2014	44.90	43.98	45.82
Unknown	2014	19.25	18.77	19.75
White	2014	71.62	71.34	71.91
Asian	2015	30.99	30.02	31.99
Black	2015	60.16	58.98	61.36
Mixed	2015	30.59	29.09	32.16
Other	2015	38.64	37.48	39.83
South Asian	2015	41.33	40.48	42.19
Unknown	2015	17.68	17.22	18.15
White	2015	66.91	66.64	67.18
Asian	2016	30.13	29.21	31.07
Black	2016	57.27	56.16	58.39
Mixed	2016	28.33	26.94	29.78
Other	2016	36.05	34.97	37.16
South Asian	2016	38.04	37.25	38.84
Unknown	2016	17.90	17.44	18.36
White	2016	66.49	66.23	66.76
Asian	2017	28.88	28.02	29.77
Black	2017	54.22	53.17	55.27
Mixed	2017	28.82	27.47	30.22
Other	2017	36.11	35.07	37.16
South Asian	2017	35.22	34.48	35.97
Unknown	2017	16.77	16.34	17.21
White	2017	63.53	63.27	63.79
Asian	2018	30.70	29.82	31.59
Black	2018	61.65	60.55	62.76
Mixed	2018	32.89	31.48	34.35
Other	2018	40.36	39.28	41.45
South Asian	2018	39.39	38.62	40.17
Unknown	2018	21.70	21.21	22.19
White	2018	80.17	79.88	80.47

Table 3: Temporal trends PSA age-standardised testing rates by year and IMD quintile per 1000 person-years between 2000 and 2018 (Data from Figure 2)

IMD Quintile	***Year***	***Standardised rate***	***Lower CI 95%***	***Upper CI 95%***

1	2000	18.77	18.43	19.11
2	2000	15.16	14.86	15.47
3	2000	12.03	11.77	12.31
4	2000	8.11	7.90	8.32
5	2000	5.64	5.46	5.82
1	2001	28.15	27.74	28.56
2	2001	22.73	22.38	23.09
3	2001	18.40	18.08	18.73
4	2001	12.45	12.20	12.70
5	2001	8.76	8.54	8.98
1	2002	37.85	37.38	38.31
2	2002	30.38	29.97	30.79
3	2002	24.44	24.08	24.81
4	2002	16.43	16.15	16.72
5	2002	12.14	11.89	12.39
1	2003	47.60	47.08	48.11
2	2003	38.36	37.90	38.81
3	2003	30.86	30.45	31.27
4	2003	20.80	20.49	21.13
5	2003	16.09	15.80	16.38
1	2004	59.31	58.74	59.88
2	2004	48.68	48.17	49.19
3	2004	38.80	38.35	39.26
4	2004	27.75	27.39	28.12
5	2004	22.01	21.68	22.35
1	2005	59.66	59.10	60.23
2	2005	50.80	50.29	51.32
3	2005	41.94	41.47	42.41
4	2005	30.94	30.55	31.33
5	2005	24.94	24.59	25.30
1	2006	62.27	61.70	62.84
2	2006	53.23	52.71	53.75
3	2006	44.37	43.89	44.85
4	2006	32.74	32.34	33.13
5	2006	26.35	25.99	26.71
1	2007	66.81	66.23	67.40
2	2007	57.87	57.33	58.41
3	2007	47.93	47.43	48.42
4	2007	35.38	34.98	35.79
5	2007	28.45	28.08	28.83
1	2008	74.22	73.61	74.83
2	2008	64.65	64.10	65.22
3	2008	53.37	52.86	53.89

4	2008	39.30	38.88	39.73
5	2008	32.68	32.28	33.08
1	2009	79.08	78.46	79.71
2	2009	68.98	68.41	69.55
3	2009	58.27	57.74	58.80
4	2009	44.45	44.01	44.91
5	2009	36.06	35.64	36.48
1	2010	78.13	77.52	78.74
2	2010	68.71	68.14	69.28
3	2010	58.40	57.87	58.93
4	2010	45.26	44.81	45.71
5	2010	37.86	37.43	38.29
1	2011	79.56	78.95	80.18
2	2011	70.21	69.64	70.78
3	2011	59.90	59.37	60.43
4	2011	46.33	45.88	46.78
5	2011	39.43	38.99	39.86
1	2012	79.01	78.41	79.62
2	2012	70.87	70.31	71.44
3	2012	60.79	60.26	61.32
4	2012	48.23	47.77	48.69
5	2012	40.33	39.89	40.76
1	2013	84.58	83.96	85.20
2	2013	76.26	75.67	76.84
3	2013	66.13	65.58	66.68
4	2013	53.08	52.61	53.56
5	2013	45.02	44.56	45.48
1	2014	82.49	81.88	83.09
2	2014	74.23	73.67	74.80
3	2014	64.23	63.70	64.77
4	2014	52.24	51.78	52.71
5	2014	43.61	43.16	44.05
1	2015	78.86	78.28	79.44
2	2015	68.85	68.31	69.39
3	2015	59.84	59.33	60.35
4	2015	48.53	48.09	48.98
5	2015	39.64	39.22	40.06
1	2016	79.61	79.03	80.19
2	2016	68.24	67.72	68.78
3	2016	58.88	58.38	59.38
4	2016	46.94	46.51	47.38
5	2016	38.86	38.46	39.28
1	2017	75.52	74.96	76.07

2	2017	65.45	64.94	65.96
3	2017	56.53	56.05	57.01
4	2017	44.93	44.52	45.35
5	2017	37.21	36.82	37.61
1	2018	96.99	96.36	97.62
2	2018	82.53	81.96	83.10
3	2018	70.22	69.69	70.76
4	2018	54.34	53.89	54.80
5	2018	44.37	43.94	44.81

Table 4: Temporal trends PSA age-standardised testing rates by year and region per 1000 person-years between 2000 and 2018 (Data from Figure 2)

Region	***Year***	***Standardised rate***	***Lower CI***	***Upper CI***
East Midlands	2000	11.65	10.95	12.39
East Midlands	2001	19.53	18.65	20.45
East Midlands	2002	25.85	24.84	26.90
East Midlands	2003	30.80	29.70	31.93
East Midlands	2004	43.73	42.41	45.09
East Midlands	2005	41.76	40.48	43.08
East Midlands	2006	41.70	40.43	43.01
East Midlands	2007	49.44	48.07	50.83
East Midlands	2008	52.09	50.71	53.51
East Midlands	2009	55.83	54.41	57.28
East Midlands	2010	54.30	52.92	55.71
East Midlands	2011	54.53	53.16	55.93
East Midlands	2012	56.11	54.73	57.51
East Midlands	2013	60.08	58.67	61.53
East Midlands	2014	59.49	58.10	60.90
East Midlands	2015	56.61	55.29	57.96
East Midlands	2016	56.72	55.42	58.05
East Midlands	2017	57.04	55.76	58.35
East Midlands	2018	69.52	68.11	70.96
East of England	2000	19.56	18.82	20.32
East of England	2001	28.61	27.74	29.50
East of England	2002	33.60	32.67	34.55
East of England	2003	37.38	36.41	38.37
East of England	2004	41.83	40.81	42.86
East of England	2005	48.06	46.99	49.16
East of England	2006	47.91	46.84	48.99
East of England	2007	53.14	52.03	54.26
East of England	2008	60.14	58.98	61.33
East of England	2009	65.47	64.27	66.69

East of England	2010	66.81	65.60	68.03
East of England	2011	65.46	64.28	66.66
East of England	2012	70.15	68.94	71.38
East of England	2013	77.68	76.41	78.96
East of England	2014	75.95	74.71	77.21
East of England	2015	71.29	70.10	72.49
East of England	2016	70.02	68.86	71.19
East of England	2017	67.34	66.22	68.48
East of England	2018	83.44	82.18	84.71
London	2000	8.52	8.30	8.74
London	2001	12.66	12.40	12.92
London	2002	15.35	15.07	15.64
London	2003	17.69	17.39	18.00
London	2004	24.52	24.17	24.88
London	2005	27.90	27.52	28.28
London	2006	31.98	31.58	32.39
London	2007	34.81	34.39	35.23
London	2008	39.96	39.51	40.41
London	2009	46.65	46.16	47.13
London	2010	48.65	48.16	49.14
London	2011	50.26	49.77	50.75
London	2012	48.45	47.97	48.93
London	2013	51.18	50.70	51.68
London	2014	52.36	51.87	52.85
London	2015	47.57	47.12	48.03
London	2016	46.24	45.81	46.68
London	2017	43.11	42.70	43.53
London	2018	49.54	49.10	49.98
North East	2000	7.65	7.13	8.21
North East	2001	15.53	14.82	16.27
North East	2002	18.17	17.41	18.97
North East	2003	22.00	21.16	22.87
North East	2004	23.53	22.67	24.43
North East	2005	27.72	26.80	28.67
North East	2006	27.80	26.89	28.74
North East	2007	28.60	27.69	29.54
North East	2008	31.57	30.62	32.55
North East	2009	33.21	32.25	34.20
North East	2010	34.56	33.59	35.56
North East	2011	37.06	36.06	38.08
North East	2012	37.37	36.38	38.38
North East	2013	43.64	42.58	44.73
North East	2014	41.58	40.55	42.63

North East	2015	39.68	38.68	40.70
North East	2016	38.99	38.01	40.00
North East	2017	37.98	37.02	38.96
North East	2018	48.37	47.29	49.47
North West	2000	9.81	9.56	10.06
North West	2001	15.47	15.16	15.78
North West	2002	21.14	20.78	21.49
North West	2003	29.35	28.94	29.77
North West	2004	40.07	39.59	40.55
North West	2005	41.58	41.10	42.06
North West	2006	42.52	42.04	43.01
North West	2007	46.90	46.40	47.41
North West	2008	52.59	52.06	53.12
North West	2009	55.65	55.11	56.19
North West	2010	56.97	56.43	57.51
North West	2011	59.36	58.81	59.91
North West	2012	61.14	60.59	61.70
North West	2013	66.98	66.41	67.56
North West	2014	63.96	63.40	64.52
North West	2015	57.44	56.92	57.96
North West	2016	57.38	56.87	57.90
North West	2017	53.11	52.62	53.60
North West	2018	64.83	64.29	65.37
South East	2000	15.11	14.81	15.41
South East	2001	23.59	23.22	23.95
South East	2002	30.43	30.02	30.84
South East	2003	40.32	39.85	40.79
South East	2004	52.26	51.74	52.80
South East	2005	52.58	52.05	53.11
South East	2006	54.77	54.24	55.30
South East	2007	57.02	56.49	57.56
South East	2008	62.92	62.36	63.47
South East	2009	68.59	68.02	69.17
South East	2010	68.18	67.62	68.75
South East	2011	68.69	68.13	69.26
South East	2012	69.67	69.11	70.24
South East	2013	75.33	74.75	75.91
South East	2014	72.49	71.92	73.05
South East	2015	68.90	68.36	69.44
South East	2016	70.55	70.01	71.09
South East	2017	68.57	68.05	69.10
South East	2018	87.42	86.82	88.02
South West	2000	18.11	17.69	18.54

South West	2001	23.90	23.43	24.38
South West	2002	32.12	31.58	32.67
South West	2003	37.48	36.90	38.07
South West	2004	42.72	42.10	43.33
South West	2005	46.73	46.09	47.37
South West	2006	49.00	48.35	49.65
South West	2007	53.60	52.94	54.28
South West	2008	60.16	59.46	60.86
South West	2009	60.57	59.88	61.28
South West	2010	59.88	59.19	60.57
South West	2011	61.07	60.38	61.76
South West	2012	62.25	61.56	62.94
South West	2013	67.02	66.31	67.73
South West	2014	67.07	66.37	67.78
South West	2015	65.79	65.10	66.49
South West	2016	61.05	60.40	61.71
South West	2017	61.32	60.67	61.97
South West	2018	78.51	77.78	79.25
West Midlands	2000	8.25	8.01	8.50
West Midlands	2001	13.65	13.35	13.96
West Midlands	2002	23.34	22.95	23.74
West Midlands	2003	32.23	31.78	32.69
West Midlands	2004	41.39	40.87	41.90
West Midlands	2005	43.77	43.25	44.30
West Midlands	2006	46.34	45.81	46.88
West Midlands	2007	49.81	49.26	50.36
West Midlands	2008	56.12	55.54	56.70
West Midlands	2009	61.81	61.21	62.41
West Midlands	2010	61.51	60.92	62.11
West Midlands	2011	63.64	63.04	64.25
West Midlands	2012	65.06	64.46	65.67
West Midlands	2013	71.55	70.92	72.18
West Midlands	2014	68.40	67.80	69.01
West Midlands	2015	63.04	62.47	63.61
West Midlands	2016	61.89	61.33	62.45
West Midlands	2017	57.92	57.39	58.46
West Midlands	2018	74.73	74.12	75.34
Yorkshire and The Humber	2000	14.41	13.73	15.14
Yorkshire and The Humber	2001	19.17	18.40	19.97
Yorkshire and The Humber	2002	24.67	23.81	25.56

Yorkshire and The Humber	2003	25.97	25.10	26.87
Yorkshire and The Humber	2004	31.45	30.50	32.42
Yorkshire and The Humber	2005	34.92	33.94	35.93
Yorkshire and The Humber	2006	36.89	35.89	37.91
Yorkshire and The Humber	2007	41.99	40.94	43.07
Yorkshire and The Humber	2008	44.88	43.80	45.98
Yorkshire and The Humber	2009	51.02	49.88	52.18
Yorkshire and The Humber	2010	47.04	45.95	48.14
Yorkshire and The Humber	2011	48.22	47.13	49.34
Yorkshire and The Humber	2012	48.03	46.94	49.13
Yorkshire and The Humber	2013	53.51	52.38	54.67
Yorkshire and The Humber	2014	50.98	49.89	52.09
Yorkshire and The Humber	2015	49.39	48.34	50.46
Yorkshire and The Humber	2016	52.19	51.12	53.28
Yorkshire and The Humber	2017	50.14	49.11	51.19
Yorkshire and The Humber	2018	64.67	63.50	65.86

Table 5: Temporal trends PSA age-standardised testing rates by year and PSA value above or below the age-specific threshold per 1000 person-years between 2000 and 2018 (Data from Figure 2)

Above or below the age-specific threshold	***Year***	***Standardised rate***	***Lower CI***	***Upper CI***
Above the age-specific threshold	2000	2.53	2.47	2.58
Above the age-specific threshold	2001	3.88	3.81	3.94
Above the age-specific threshold	2002	5.17	5.10	5.25
Above the age-specific threshold	2003	6.68	6.59	6.76
Above the age-specific threshold	2004	8.31	8.21	8.40

Above the age-specific threshold	2005	8.54	8.45	8.64
Above the age-specific threshold	2006	9.40	9.31	9.50
Above the age-specific threshold	2007	9.85	9.75	9.95
Above the age-specific threshold	2008	10.12	10.02	10.22
Above the age-specific threshold	2009	10.85	10.75	10.95
Above the age-specific threshold	2010	11.04	10.94	11.14
Above the age-specific threshold	2011	11.47	11.36	11.57
Above the age-specific threshold	2012	11.58	11.48	11.69
Above the age-specific threshold	2013	12.01	11.91	12.12
Above the age-specific threshold	2014	11.58	11.48	11.68
Above the age-specific threshold	2015	11.06	10.96	11.15
Above the age-specific threshold	2016	10.77	10.68	10.87
Above the age-specific threshold	2017	10.44	10.35	10.54
Above the age-specific threshold	2018	11.89	11.80	11.99
Below the age-specific threshold	2000	9.30	9.20	9.40
Below the age-specific threshold	2001	14.04	13.92	14.16
Below the age-specific threshold	2002	18.85	18.71	19.00
Below the age-specific threshold	2003	23.80	23.64	23.95
Below the age-specific threshold	2004	30.75	30.57	30.92
Below the age-specific threshold	2005	32.89	32.71	33.08
Below the age-specific threshold	2006	34.19	34.01	34.38
Below the age-specific threshold	2007	37.29	37.10	37.48
Below the age-specific threshold	2008	42.60	42.39	42.80
Below the age-specific threshold	2009	46.43	46.22	46.64
Below the age-specific threshold	2010	46.55	46.35	46.76

Below the age-specific threshold	2011	47.54	47.33	47.75
Below the age-specific threshold	2012	48.23	48.02	48.44
Below the age-specific threshold	2013	52.98	52.77	53.20
Below the age-specific threshold	2014	51.78	51.57	52.00
Below the age-specific threshold	2015	48.11	47.91	48.31
Below the age-specific C threshold	2016	47.76	47.57	47.96
Below the age-specific threshold	2017	45.50	45.31	45.69
Below the age-specific threshold	2018	57.83	57.62	58.05

Caption: PSA values were categorised as above the age specific threshold if patients were aged 18-49 years with a PSA value >2.5 ng/mL, 50-59 years with a PSA value >3.5 ng/mL, 60-69 years with a PSA value >4.5 ng/mL, and ≥70 with a PSA value >6.5 ng/mL

Table 6: Temporal trends PSA age-standardised testing rates by year and symptom presentation per 1000 person-years between 2000 and 2018 (Data from Figure 2)

Symptom present 90 days before PSA test (Yes/No)	***Year***	***Standardised rate***	***Lower CI***	***Upper CI***
Symptoms present	2000	3.33	3.26	3.39
No symptoms present	2000	8.50	8.40	8.60
Symptoms present	2001	4.91	4.84	4.99
No symptoms present	2001	13.00	12.88	13.12
Symptoms present	2002	6.58	6.49	6.66
No symptoms present	2002	17.45	17.31	17.59
Symptoms present	2003	8.21	8.11	8.30
No symptoms present	2003	22.27	22.12	22.42
Symptoms present	2004	10.15	10.05	10.25
No symptoms present	2004	28.90	28.73	29.08
Symptoms present	2005	11.13	11.03	11.24
No symptoms present	2005	30.30	30.13	30.48
Symptoms present	2006	11.51	11.40	11.62
No symptoms present	2006	32.09	31.91	32.27
Symptoms present	2007	12.59	12.48	12.71

No symptoms present	2007	34.54	34.36	34.73
Symptoms present	2008	13.64	13.53	13.76
No symptoms present	2008	39.08	38.88	39.27
Symptoms present	2009	14.85	14.73	14.97
No symptoms present	2009	42.42	42.22	42.62
Symptoms present	2010	15.36	15.24	15.48
No symptoms present	2010	42.24	42.04	42.44
Symptoms present	2011	16.00	15.87	16.12
No symptoms present	2011	43.01	42.82	43.21
Symptoms present	2012	16.23	16.11	16.35
No symptoms present	2012	43.58	43.38	43.78
Symptoms present	2013	18.36	18.23	18.49
No symptoms present	2013	46.63	46.43	46.84
Symptoms present	2014	17.41	17.29	17.53
No symptoms present	2014	45.96	45.76	46.16
Symptoms present	2015	15.92	15.80	16.03
No symptoms present	2015	43.25	43.06	43.44
Symptoms present	2016	15.68	15.57	15.79
No symptoms present	2016	42.86	42.67	43.05
Symptoms present	2017	14.98	14.87	15.09
No symptoms present	2017	40.97	40.79	41.15
Symptoms present	2018	17.92	17.80	18.05
No symptoms present	2018	51.80	51.60	52.00

Table 7: Temporal trends PSA age-standardised testing rates by year and family history of prostate cancer per 1000 person-years between 2000 and 2018 (Data from Figure 2)

Family history of prostate cancer	*Year*	*Standardised rate*	*Lower CI*	*Upper CI*
No family history	2000	11.77	11.65	11.89
Family history	2000	42.52	33.69	55.02
No family history	2001	17.83	17.69	17.97
Family history	2001	50.59	42.46	61.36
No family history	2002	23.90	23.74	24.06
Family history	2002	72.45	63.90	82.86
No family history	2003	30.31	30.13	30.49

Family history	2003	89.90	81.04	99.99
No family history	2004	38.83	38.63	39.03
Family history	2004	117.36	108.06	127.59
No family history	2005	41.14	40.93	41.35
Family history	2005	151.36	138.82	170.89
No family history	2006	43.25	43.04	43.46
Family history	2006	162.47	152.83	175.69
No family history	2007	46.75	46.54	46.97
Family history	2007	166.97	158.12	177.49
No family history	2008	52.22	52.00	52.45
Family history	2008	196.51	187.66	206.42
No family history	2009	56.72	56.49	56.95
Family history	2009	204.39	196.15	213.14
No family history	2010	57.04	56.80	57.27
Family history	2010	194.28	186.84	202.11
No family history	2011	58.37	58.13	58.60
Family history	2011	208.23	200.97	215.76
No family history	2012	59.12	58.89	59.35
Family history	2012	211.52	204.60	218.66
No family history	2013	64.13	63.89	64.37
Family history	2013	242.31	235.33	249.47
No family history	2014	62.48	62.25	62.72
Family history	2014	236.68	230.12	243.41
No family history	2015	58.26	58.04	58.48
Family history	2015	230.57	224.38	236.91
No family history	2016	57.55	57.33	57.77
Family history	2016	237.26	231.27	243.37
No family history	2017	54.89	54.68	55.11
Family history	2017	239.37	233.57	245.28
No family history	2018	68.34	68.11	68.58
Family history	2018	305.92	299.44	312.50
