# Supplementary material for: Prostate specific antigen retesting intervals and trends in England: population based cohort study
Source: BMJ. 2025 Oct 8;391:e083800. doi: 10.1136/bmj-2024-083800 (PMC12505835; doi:10.1136/bmj-2024-083800)
Supplement: Supplementary file 3 — Web appendix 3: Additional file 3 [file colk083800.ww3.pdf]

Additional file 3

Sensitivity analysis 1

The impact on PSA retesting intervals when including PSA tests that were more than one month apart to remove bias from having a repeat PSA test to confirm first raised PSA

N = 720,403 distinct patients and 2,228,291 intervals between PSA tests

Table 1: Linear mixed effect models for length of PSA retesting intervals: Multivariable Models*

	**Interval ratios**	**95% CI**	**Expected months**	**P value**
**Intercept***	18.38	(18.08–18.68)		<0.001
**Region (ref South East)**				<0.001
East Midlands	0.97	(0.93–1.01)	17.76	
East of England	1.02	(0.98–1.06)	18.70	
London	1.02	(1.00–1.04)	18.74	
North East	1.09	(1.05–1.13)	20.04	
North West	0.98	(0.96–1.00)	18.05	
South West	0.96	(0.94–0.99)	17.70	
West Midlands	1.03	(1.00–1.05)	18.87	
Yorkshire & Humber	0.98	(0.94–1.02)	18.07	
Unknown	0.87	(0.69–1.09)	16.00	
**Age range (ref 60–69)**				<0.001
18–29	0.51	(0.47–0.55)	9.28	
30–39	0.72	(0.70–0.73)	13.13	
40–49	0.96	(0.96–0.97)	17.72	
50–59	1.04	(1.04–1.05)	19.19	
70–79	0.93	(0.92–0.93)	17.02	
80–89	0.97	(0.97–0.98)	17.84	
90+	1.11	(1.09–1.12)	20.35	
**Ethnicity (ref White)**				<0.001
Asian	0.95	(0.94–0.97)	17.54	
Black	0.97	(0.96–0.98)	17.78	
Mixed	0.99	(0.96–1.02)	18.17	
Other	0.98	(0.97–1.00)	18.05	
South Asian	0.97	(0.96–0.98)	17.76	
Unknown	0.89	(0.88–0.90)	16.33	
**IMD (ref 1)**				<0.001
2	1.00	(0.99–1.00)	18.38	
3	1.00	(0.99–1.00)	18.32	
4	0.99	(0.98–1.00)	18.22	
5	0.99	(0.98–1.00)	18.17	
Unknown	1.03	(0.97–1.10)	18.95	
**PSA above threshold**				<0.001
Yes	0.59	(0.59–0.60)	10.92	
**Family history**				<0.001
Yes	1.01	(0.99–1.02)	18.46	
**Symptoms (ref No)**				
Fatigue	1.22	(1.20–1.23)	22.32	

Bone Pain	1.12	(1.07–1.16)	20.51	
Back Pain	1.19	(1.18–1.19)	21.77	<0.001
UWL	1.18	(1.16–1.20)	21.70	<0.001
Haematuria	1.20	(1.19–1.21)	22.11	<0.001
ED	1.18	(1.17–1.19)	21.74	<0.001
LUTS	1.26	(1.25–1.26)	23.08	<0.001

“Months” refers to the estimated geometric mean time between repeat PSA tests. The intercept represents the mean interval for the reference group (age 60-90, white, IMD1, South East, no symptoms, no raised PSA, no family history). For each covariate level, values were calculated by multiplying the reference interval by the exponentiated fixed effect, giving the estimated interval in months while holding all other covariates at their reference level.

Findings: When restricting the analysis to PSA tests conducted at least one month apart (to remove the influence of repeat testing performed to confirm an initial elevated result), the impact on retesting intervals was most pronounced for PSA levels themselves. In this analysis, having a previous elevated PSA reduced the interval by approximately 7.5 months, compared to a 13-month reduction observed in the main analysis. Age also showed some differences, although the overall patterns and direction of associations remained consistent.

Sensitivity analysis 2

The impact of restricting to patients with over six years of follow-up to mitigate potential censoring bias. We ran analysis on cohorts who had more than six years of follow up from study entry to exit.

N = 660,312 distinct patients and 2,176,547 retesting intervals

Table 2: Linear mixed effect models for length of PSA retesting intervals: Multivariable Models*

	**Interval ratios**	**95% CI**	**Expected months**	**P value**
**Intercept***	20.00	(19.60–20.40)		
Region (ref South East)				<0.001
**East Midlands**	0.98	(0.93–1.03)	19.5	
**East of England**	1.03	(0.98–1.07)	20.5	
**London**	0.99	(0.97–1.02)	19.8	
**North East**	1.04	(0.99–1.08)	20.7	
**North West**	0.95	(0.92–0.97)	18.9	
**South West**	1.02	(0.99–1.05)	20.4	
**Unknown**	0.97	(0.75–1.25)	19.3	
**West Midlands**	1.04	(1.01–1.07)	20.8	
**Yorkshire & Humber**	0.97	(0.93–1.02)	19.5	
IMD (ref 1)				<0.001
**2**	1.00	(0.99–1.01)	20.0	
**3**	1.00	(0.99–1.00)	20.0	
**4**	0.99	(0.98–1.00)	19.8	
**5**	0.99	(0.98–1.00)	19.7	
**Unknown**	1.01	(0.94–1.08)	20.2	
Ethnicity (ref White)				<0.001

**Asian**	0.90	(0.88–0.91)	17.9	
**Black**	0.90	(0.89–0.91)	18.0	
**Mixed**	0.89	(0.86–0.91)	17.7	
**Other**	0.91	(0.89–0.93)	18.2	
**South Asian**	0.91	(0.90–0.92)	18.2	
**Unknown**	0.93	(0.92–0.94)	18.6	
Age range (ref 60–69)				<0.001
**18–29**	0.94	(0.87–1.01)	18.8	
**30–39**	1.57	(1.53–1.60)	31.4	
**40–49**	1.56	(1.55–1.58)	31.2	
**50–59**	1.27	(1.27–1.28)	25.4	
**70–79**	0.76	(0.76–0.77)	15.3	
**80–89**	0.68	(0.67–0.68)	13.5	
**90+**	0.56	(0.55–0.57)	11.3	
Family history				<0.001
**Yes**	0.84	(0.82–0.85)	16.7	
PSA value above age-specific threshold				<0.001
**Yes**	0.33	(0.33 – 0.33)	6.6	
Symptoms				
**Fatigue**	1.00	(0.99–1.01)	19.9	
**Haematuria**	0.95	(0.94–0.96)	19.0	<0.001
**Bone Pain**	0.99	(0.93–1.04)	19.7	
**Back Pain**	1.04	(1.03–1.05)	20.7	<0.001
**UWL**	0.97	(0.95–0.99)	19.4	<0.001
**ED**	1.05	(1.04–1.06)	20.9	<0.001
**LUTS**	1.03	(1.02 – 1.03)	20.5	<0.001

"Months" refers to the estimated geometric mean time between repeat PSA tests. The intercept represents the mean interval for the reference group (age 60-90, white, IMD1, South East, no symptoms, no raised PSA, no family history). For each covariate level, values were calculated by multiplying the reference interval by the exponentiated fixed effect, giving the estimated interval in months while holding all other covariates at their reference level.

Findings: The intercept is over a month longer than in the overall cohort, while all other model results and directions remain similar.
