# Supplementary material for: Prostate specific antigen retesting intervals and trends in England: population based cohort study
Source: BMJ. 2025 Oct 8;391:e083800. doi: 10.1136/bmj-2024-083800 (PMC12505835; doi:10.1136/bmj-2024-083800)
Supplement: Supplementary file 4 — Web appendix 4: Additional file 4 [file colk083800.ww4.pdf]

Additional File 4

SNOMED and READCODE list for Prostate Specific Antigen (PSA) test in CPRD Aurum

Term	Snomed CT ConceptId	Read Code	Test
Prostate specific antigen normal	166159005	43Z2000	PSA
Prostate specific antigen abnormal	166160000	43Z2100	PSA
PSA - Serum prostate specific antigen level	1030791000000100	43Z2.11	PSA
Raised PSA	396152005	R15y011	PSA
PSA (prostate-specific antigen) level	1030791000000100	43Z2.00	PSA
Raised prostate specific antigen	396152005	R15y000	PSA
Ultrasensitive prostate specific antigen level	443174008	43ZG.00	PSA
PSA (prostate specific antigen) monitored in primary care	1822111000006103	NULL	PSA
Prostate-specific antigen threshold for referral	1890971000006104	NULL	PSA
Serum PSA (prostate specific antigen) level	1000381000000105	43Z2200	PSA
Prostate specific antigen threshold for referral	920411000000105	66Z0.00	PSA
Prostate specific antigen measurement	63476009	NULL	PSA
PSA measurement	63476009	NULL	PSA
PSA - Serum prostate specific antigen level	63476009	NULL	PSA
PSA - Prostate-specific antigen level	63476009	NULL	PSA
Prostate specific antigen	102687007	NULL	PSA
PSA - Prostate specific antigen	102687007	NULL	PSA
Elevated PSA	396152005	NULL	PSA
Serum prostate specific antigen measurement	909321000000105	NULL	PSA
Serum prostate specific antigen level	909321000000105	NULL	PSA
Prostate specific antigen level	1030791000000100	NULL	PSA

SNOMED and READCODE list for symptoms in CPRD Aurum

Term	Snomed CT ConceptId	Read Code	Symptom group
Acute back pain - unspecified	161891005	N145.11	Back Pain
Acute back pain - lumbar	278862001	N142.13	Back Pain
[X]Other dorsalgia	410730009	Nyu7A00	Back Pain
Acute low back pain	278862001	N12..13	Back Pain
Acute back pain with sciatica	247366003	N143.11	Back Pain
Backache	161891005	16C2.00	Back Pain
Back pain	161891005	N145.12	Back Pain
Arthralgia of sacroiliac joint	202487003	N094L00	Back Pain
Acute thoracic back pain	279035001	N141.11	Back Pain
Backache NOS	643261000000101	N145.99	Back Pain
Backache symptom	161891005	16C..00	Back Pain
Back pain worse on sneezing	161893008	16C4.00	Back Pain
Back pain without radiation NOS	161891005	16C6.00	Back Pain
Backache with radiation	161892003	16C3.00	Back Pain
Backache symptom NOS	161891005	16CZ.00	Back Pain
C/O - low back pain	161894002	16C5.00	Back Pain
C/O - lumbar pain	161894002	1A53.12	Back Pain
Chronic low back pain	278860009	16C9.00	Back Pain
Backache, unspecified	161891005	N145.00	Back Pain
Coccygodynia	34789001	N147200	Back Pain
C/O - upper back ache	161896000	16C7.00	Back Pain
Low back pain	279039007	N142.11	Back Pain
Exacerbation of backache	135860001	16C8.00	Back Pain
Low back pain clinical pathway	847561000000101	8CMW000	Back Pain
Chronic back pain	134407002	NULL	Back Pain
Other cervical syndromes	410730009	N13y.00	Back Pain
Facet joint syndrome	247369005	N14y.11	Back Pain
Non-mechanical back pain	1573621000006105	NULL	Back Pain
Disorder of spinal region	410730009	N14z.12	Back Pain
Pain in cervical spine	279029001	N131.11	Back Pain
Lumbago with sciatica	202794004	N142000	Back Pain
Pain in lumbar spine	267982002	N142.00	Back Pain
Low back pain clinical pathway protocol not followed	1824111000006107	NULL	Back Pain
Pain in coccyx	34789001	N147211	Back Pain
Lumbalgia	279039007	N142.12	Back Pain

Thoracic back pain	279038004	NULL	Back Pain
Lumbago	279039007	N142.14	Back Pain
Pain in thoracic spine	267981009	N141.00	Back Pain
Mechanical low back pain	279040009	16CA.00	Back Pain
Prolapsed lumbar intervertebral disc with sciatica	311804006	N12C400	Back Pain
O/E - lumbar pain on palpation	275316003	25C..13	Back Pain
Referral to back pain clinic	183868005	8HTH.00	Back Pain
STarT back score - bothered by back pain last 2wks	1916461000006107	NULL	Back Pain
Sciatica	23056005	N143.00	Back Pain
Other specified disorders of vertebral column	410730009	N1y..00	Back Pain
Vertebral column disorder NOS	410730009	N1z..00	Back Pain
STarT back score - back pain down leg(s) in last 2 weeks	1916371000006108	NULL	Back Pain
Thoracic nerve root pain	103015000	N144011	Back Pain
Rib pain	297217002	182B.00	Bone Pain
Bony pelvic pain	203508001	N33A000	Bone Pain
Bone tenderness	278997003	NULL	Bone Pain
Bone pain	12584003	N33A.00	Bone Pain
Costal margin chest pain	161977000	182B000	Bone Pain
Clavicle pain	203509009	N33A100	Bone Pain
Cannot sustain an erection	248779006	1ABC.00	ED
Erectile dysfunction	397803000	E227311	ED
[X]Male erectile disorder	397803000	Eu52212	ED
Advice about impotence	135885005	67IA.00	ED
Erectile dysfunction organic	198036002	K27y199	ED
Erectile dysfunction due to diabetes mellitus	867891000000101	K27y700	ED
Impotence of organic origin	198036002	K27y100	ED
C/O erectile dysfunction	473327001	1D1B.00	ED
Impotence	397803000	E227300	ED
Referral to erectile dysfunction clinic	704048004	8HTj.00	ED
Psychogenic impotence	73491007	Eu52213	ED
Cannot get an erection	248773007	1ABB.00	ED
Penile injection to produce erection	176566004	7C25B00	ED

Diabetic erectile dysfunction review	473209001	66Au.00	ED
Referral to erectile dysfunction clinic declined	704049007	8IE8.00	ED
Management of erectile dysfunction	851081000000105	7C25E00	ED
[X]Neurasthenia	78667006	Eu46000	Fatigue
[D]Lethargy	214264003	R007300	Fatigue
[D]Fatigue	84229001	R007100	Fatigue
[D]Malaise and fatigue	271795006	R007.00	Fatigue
Chronic fatigue syndrome	52702003	F286.00	Fatigue
[X]Fatigue syndrome	84229001	Eu46011	Fatigue
[D]Tiredness	267031002	R007500	Fatigue
[D]Malaise and fatigue NOS	271795006	R007z00	Fatigue
Depressive personality	78667006	E211299	Fatigue
Asthenia	13791008	R007200	Fatigue
C/O - debility - malaise	272036004	1684.11	Fatigue
[X]Depressive neurosis	78667006	Eu34111	Fatigue
Malaise	367391008	R007000	Fatigue
C/O - 'tired all the time'	272062008	1683.11	Fatigue
Depressive neurosis	78667006	Eu34113	Fatigue
CFS - Chronic fatigue syndrome	52702003	F286.11	Fatigue
Myalgic encephalomyelitis	52702003	F03y.12	Fatigue
Debility	13791008	R2y3.00	Fatigue
Depressive personality disorder	78667006	E211200	Fatigue
Exhaustion	60119000	1688.00	Fatigue
Myalgic encephalomyelitis	52702003	F286.15	Fatigue
Fatigue	84229001	1682.00	Fatigue
Dysthymia	78667006	Eu34100	Fatigue
Fatigue - symptom	272060000	168..11	Fatigue
Neurasthenia	78667006	E205.00	Fatigue
Fatigue fracture of vertebra	202831001	N1y1.00	Fatigue
Nervous debility - neurasthenia	52702003	E205.99	Fatigue
Lethargy	214264003	168..12	Fatigue
Referral for chronic fatigue syndrome activity management	522991000000100	8HlL.00	Fatigue
Feels unwell	367391008	16E..00	Fatigue
PHQ9 score - feels tired	1008801000006103	NULL	Fatigue
Malaise - symptom	367391008	168..13	Fatigue

Referral to chronic fatigue syndrome specialist team	520511000000101	8HkW.00	Fatigue
Lassitude	13791008	R007z11	Fatigue
Tiredness symptom NOS	267031002	168Z.00	Fatigue
Mild chronic fatigue syndrome	377181000000104	F286000	Fatigue
Senile exhaustion	57494004	R204.00	Fatigue
Malaise and fatigue	271795006	1684.00	Fatigue
Somatic complaints/fatigue	959231000006104	NULL	Fatigue
Moderate chronic fatigue syndrome	377171000000101	F286100	Fatigue
Tiredness symptom	267031002	168..00	Fatigue
ME - Myalgic encephalomyelitis	52702003	F286.16	Fatigue
Severe chronic fatigue syndrome	377161000000108	F286200	Fatigue
Nervous exhaustion	78667006	E205.11	Fatigue
Tired all the time	267032009	1683.00	Fatigue
Sick	367391008	16E..12	Fatigue
Tired all the time	267032009	E205.12	Fatigue
Transient heat fatigue	12979003	SN26.00	Fatigue
Weakness - general	13791008	R007211	Fatigue
Blood in urine - symptom	53298000	1A45.11	Haematuria
Clot haematuria	197942003	K197400	Haematuria
Haematuria NOS	34436003	K197.99	Haematuria
Blood in urine	34436003	46G2.11	Haematuria
Recurrent haematuria	281860005	K0A2.00	Haematuria
H/O: haematuria	161550001	14D5.00	Haematuria
Microscopic haematuria	197940006	K197200	Haematuria
Blood in urine - haematuria	53298000	1A45.00	Haematuria
Referral to haematuria clinic	700432001	8T10.00	Haematuria
Urine blood test = +	167300001	4695.00	Haematuria
Painless haematuria	197938001	K197000	Haematuria
Essential haematuria	314280007	K197.12	Haematuria
Recurrent benign haematuria syndrome	197627003	K032100	Haematuria
Frank haematuria	197941005	K197300	Haematuria
Seen in haematuria clinic	1682771000006107	NULL	Haematuria
Haematuria	34436003	K197.00	Haematuria
Urine blood test = +++	167302009	4697.00	Haematuria
Haematuria - symptom	34436003	1A45.12	Haematuria
Urine: red - blood	167235001	4625.00	Haematuria
Haematuria/abnormal colour	962031000006109	NULL	Haematuria
Painful haematuria	197939009	K197100	Haematuria

Referred to haematuria clinic	1682781000006105	NULL	Haematuria
Urine blood test = ++	167301002	4696.00	Haematuria
[D] Urgency of micturition	75088002	R086200	LUTS Overall
[D]Painful urination	49650001	R081000	LUTS Overall
[D]Dysuria NOS	49650001	R081z00	LUTS Overall
[D] Urge incontinence	87557004	R083200	LUTS Overall
[D]Incontinence of urine	165232002	R083.00	LUTS Overall
[D]Retention of urine unspecified	267064002	R082400	LUTS Overall
[D]Incontinence of urine NOS	165232002	R083z00	LUTS Overall
[D]Difficulty with micturition	102835006	R086.11	LUTS Overall
[X]Other difficulties with micturition	102835006	Ryu4000	LUTS Overall
[D]Urethral sphincter incontinence	165232002	R083100	LUTS Overall
[RFC] Urinary incontinence	908921000006109	NULL	LUTS Overall
[D]Dysuria	49650001	R081.00	LUTS Overall
[X]Psychogenic dysuria	231517009	Eu45319	LUTS Overall
[D]Urinary system symptoms NOS	249274008	R08zz00	LUTS Overall
[X]Psychogenic dyspepsia	231517009	Eu45318	LUTS Overall
[D]Frequency of micturition or polyuria NOS	274734008	R084z00	LUTS Overall
Abnormal urination	38671000119103	R086.00	LUTS Overall
[RFC] Incontinence both urinary & bowel	908931000006107	NULL	LUTS Overall
Abnormal urinary stream	84800009	NULL	LUTS Overall
[D]Nocturia	139394000	R084200	LUTS Overall
Chronic retention of urine	236650000	R082300	LUTS Overall
Bladder: incontinent	165232002	3940.00	LUTS Overall
Acute retention of urine	236648008	R082200	LUTS Overall
[D]Polyuria	28442001	R084100	LUTS Overall
Degree of urinary incontinence: moderate	1936541000006102	NULL	LUTS Overall
Continence care	183001000	8C14.11	LUTS Overall
Bladder: urge incontinence	917531000006102	NULL	LUTS Overall
[D]Urination abnormality NOS	38671000119103	R086z00	LUTS Overall
Double incontinence	78459008	16F..00	LUTS Overall
Dribbling of urine	58972000	1A37.00	LUTS Overall

Burning/painful urination	962001000006101	NULL	LUTS Overall
[X]Psychogenic IBS	231517009	Eu45324	LUTS Overall
Frequency of micturition	300471006	1A1..11	LUTS Overall
Dysuria	49650001	1A55.00	LUTS Overall
Clot retention of urine	236651001	R082000	LUTS Overall
Bedwetting	8009008	1A22011	LUTS Overall
Functional urinary and faecal incontinence	925701000000107	16F0.00	LUTS Overall
Functional urinary incontinence	129847007	1A23000	LUTS Overall
Genitourinary symptom NOS	267062003	1AZZ.00	LUTS Overall
Cannot pass urine - retention	267064002	1A32.00	LUTS Overall
Hesitancy	5972002	1A34.00	LUTS Overall
GU symptoms	267062003	1A...11	LUTS Overall
Genitourinary symptoms	267062003	1A...00	LUTS Overall
Degree of urinary incontinence	1936521000006109	NULL	LUTS Overall
Incontinence of urine	165232002	1A23.00	LUTS Overall
Hesitancy of micturition	5972002	1A34.11	LUTS Overall
Genitourinary symptoms NOS	267062003	1AZ..00	LUTS Overall
Degree of urinary incontinence: mild	1936531000006107	NULL	LUTS Overall
Lower urinary tract symptoms	307541003	1AZ6.00	LUTS Overall
Incomplete emptying of bladder	249288007	1AH0.00	LUTS Overall
Incontinence control	266826009	8D71.00	LUTS Overall
Difficulty with micturition	102835006	1AZ3.00	LUTS Overall
Micturition frequency NOS	300471006	1A1Z.00	LUTS Overall
Increased frequency of urination	162116003	R084000	LUTS Overall
LUTS - Lower urinary tract symptoms	1726491000006101	NULL	LUTS Overall
Enuresis	8009008	1A22.00	LUTS Overall
Micturition stream poor	162128006	1A33.00	LUTS Overall
Micturition frequency and polyuria	274734008	R084.00	LUTS Overall
Micturition control	129009001	1A2Z.00	LUTS Overall
Finding of frequency of urination	300471006	NULL	LUTS Overall
Mixed urinary incontinence	413343005	NULL	LUTS Overall
Mild lower urinary tract symptoms	763121000000102	1AZ6000	LUTS Overall

Nocturia	917591000006103	NULL	LUTS Overall
Frequency of micturition	300471006	1A12.00	LUTS Overall
Observation of frequency of urination	300471006	1A1..00	LUTS Overall
Referral to incontinence clinic	415274007	8HTX.00	LUTS Overall
Pis en deux	299271000000100	1A27.11	LUTS Overall
Incontinence care	183001000	8C14.00	LUTS Overall
Overflow incontinence of urine	397878005	NULL	LUTS Overall
Severe lower urinary tract symptoms	763101000000106	1AZ6200	LUTS Overall
Polyuria	28442001	1A1..12	LUTS Overall
Incontinence control	266826009	8D7..12	LUTS Overall
Painful micturition	49650001	Ryu4200	LUTS Overall
Stress incontinence	22220005	1A24.00	LUTS Overall
Poor flow of urine	162128006	NULL	LUTS Overall
IPSS - nocturia	759421000006108	NULL	LUTS Overall
Poor stream of urine	162128006	R086300	LUTS Overall
Terminal dribbling of urine	162130008	1A36.00	LUTS Overall
Precipitancy	75088002	1A35.00	LUTS Overall
LUTS - Lower urinary tract symptoms	307541003	NULL	LUTS Overall
Referred for provision of incontinence pad	1937461000006101	NULL	LUTS Overall
Urgency of micturition	75088002	1A25.11	LUTS Overall
Prostatism	11441004	1AA..00	LUTS Overall
Micturition volume	251975009	1AC..00	LUTS Overall
Slowing of urinary stream	84471002	R086000	LUTS Overall
Urinary stream - intermittent	74671000006109	NULL	LUTS Overall
Prostatism	11441004	K20..16	LUTS Overall
Moderate lower urinary tract symptoms	763111000000108	1AZ6100	LUTS Overall
Somatoform autonomic dysfunction	231517009	Eu45300	LUTS Overall
Urinary symptoms	249274008	1A...12	LUTS Overall
Psychogenic dysuria	191978003	E265300	LUTS Overall
Nocturia	139394000	1A13.00	LUTS Overall
Splitting of urinary stream	28490008	R086100	LUTS Overall
Nocturia	981411000006101	NULL	LUTS Overall
Retention of urine	267064002	R082.00	LUTS Overall
Nocturnal enuresis	8009008	1A22000	LUTS Overall
Stranguria	16844001	R081100	LUTS Overall
Polyuria	28442001	1AC2.00	LUTS Overall
Stress incontinence - symptom	22220005	1A24.11	LUTS Overall

Precipitancy of micturition	75088002	1A35.11	LUTS Overall
Total urinary incontinence	129853007	NULL	LUTS Overall
Retention - symptom	267064002	1A32.11	LUTS Overall
Urge incontinence of urine	918181000006108	NULL	LUTS Overall
Strangury	16844001	1A56.00	LUTS Overall
Urge incontinence of urine	87557004	1A26.00	LUTS Overall
Stress incontinence	22220005	K198.00	LUTS Overall
Urge to pass urine again shortly after finishing voiding	299271000000100	1A27.00	LUTS Overall
Voided urinary volume	251975009	3178.00	LUTS Overall
Urinary incontinence	165232002	R083000	LUTS Overall
Urgent desire to urinate	75088002	1A25.00	LUTS Overall
Urinary frequency	162116003	1A1..13	LUTS Overall
Urinary incontinence	165232002	Kyu5A00	LUTS Overall
Urinary retention	267064002	NULL	LUTS Overall
Urinary symptom change	170877009	66K3.00	LUTS Overall
Urinary system symptoms	249274008	R08..00	LUTS Overall
[D]Abnormal loss of weight	267024001	R032.00	UWL
Complaining of weight loss	198511000000103	1D1A.00	UWL
Abnormal weight loss	267024001	1625.00	UWL
[D]Cachexia NOS	238108007	R2y4z00	UWL
Abnormal weight loss - symptom	267024001	1625.11	UWL
Unexplained/progressive weight loss	960561000006106	NULL	UWL
O/E - cachexic	162684007	2224.00	UWL
Unintentional weight loss	448765001	1627.00	UWL
Cachexia	238108007	R2y4.00	UWL
Weight decreasing	161832001	1623.00	UWL
Weight Loss	161832001	1623.99	UWL

Prostate cancer diagnosis code used in NCRAS and HES was ICD C61%

SNOMED Codes for Prostate Cancer in CRPD Aurum

Term	SnomedCTConceptId	Term
Metastatic prostate cancer	314994000	Metastatic prostate cancer
History of malignant neoplasm of prostate	428262008	History of malignant neoplasm of prostate
Cancer of prostate	399068003	Cancer of prostate

Malignant tumor of prostate	399068003	Malignant tumor of prostate
Gleason prostate grade 5-7 (medium)	394932008	Gleason prostate grade 5-7 (medium)
Prostate carcinoma	254900004	Prostate carcinoma
Malignant tumour of prostate	399068003	Malignant tumour of prostate
Carcinoma of prostate	254900004	Carcinoma of prostate
Malignant prostatic tumour	399068003	Malignant prostatic tumour
[V]Personal history of malignant neoplasm of prostate	428262008	[V]Personal history of malignant neoplasm of prostate
Prostate cancer	254900004	Prostate cancer
Metastasis from malignant tumour of prostate	314994000	Metastasis from malignant tumour of prostate
CA - Cancer of prostate	399068003	CA - Cancer of prostate
Gleason Score 8-10: Poorly differentiated	369777009	Gleason Score 8-10: Poorly differentiated
H/O: prostate cancer	428262008	H/O: prostate cancer
Gleason prostate grade 8-10 (high)	369777009	Gleason prostate grade 8-10 (high)
Gleason prostate grade 2-4 (low)	369775001	Gleason prostate grade 2-4 (low)
[RFC] Cancer of the prostate	906971000006106	[RFC] Cancer of the prostate

Ethnicity groups in HES

Group in HES	Final Group
NULL	Unknown
Bangladesi	South Asian
Bl_Afric	Black
Bl_Carib	Black
Bl_Other	Black
Chinese	Asian
Indian	South Asian
Mixed	Mixed
Oth_Asian	Asian
Other	Other
Pakistani	South Asian
Unknown	Unknown
White	White

Ethnicity SNOMED Codes in CPRD Aurum

medcodeid	term	snomedct_conceptid	eth 2021 categories	Final group
286020010	other asian	315280000	Any other Asian background	Asian
405069018	e afric asian/indo-carib (nmo)	270465005	Any other Asian background	Asian

141541000000115	east african asian - ethnic category 2001 census	92661000000108	Any other Asian background	Asian
141521000000110	punjabi - ethnic category 2001 census	92641000000107	Any other Asian background	Asian
141571000000114	caribbean asian - ethnic category 2001 census	92691000000102	Any other Asian background	Asian
1968341000006116	asian/asian british: other asian - ni ethnic cat 2011 census	977771000000105	Any other Asian background	Asian
141381000000117	other asian background - ethnic category 2001 census	92481000000101	Any other Asian background	Asian
937651000006117	other asian or asian unspecified ethnic category 2001 census	92701000000102	Any other Asian background	Asian
411584018	indo-caribbean (nmo)	275597009	Any other Asian background	Asian
411583012	east african asian (nmo)	275596000	Any other Asian background	Asian
136081000000111	sri lankan - ethnic category 2001 census	86461000000107	Any other Asian background	Asian
1968171000006110	asian/asian brit: other asian-eng+wales eth cat 2011 census	976871000000103	Any other Asian background	Asian
141531000000112	kashmiri - ethnic category 2001 census	92651000000105	Any other Asian background	Asian
285977016	other asian (nmo)	186013000	Any other Asian background	Asian
459784018	other asian ethnic group	315281001	Any other Asian background	Asian
141551000000117	tamil - ethnic category 2001 census	92671000000101	Any other Asian background	Asian
157301000000116	sinhalese - ethnic category 2001 census	110781000000102	Any other Asian background	Asian
285976013	indian sub-continent (nmo)	186012005	Any other Asian background	Asian
1968521000006118	asian: other asian group - scotland ethnic cat 2011 census	978211000000108	Any other Asian background	Asian

141401000000117	chinese - ethnic category 2001 census	92511000000107	Chinese	Asian
1968161000006115	asian/asian brit: chinese - eng+wales ethnic cat 2011 census	976851000000107	Chinese	Asian
1968331000006114	asian/asian british: chinese - ni ethnic cat 2011 census	977751000000101	Chinese	Asian
1968511000006114	asian: chinese - scotland ethnic category 2011 census	978191000000109	Chinese	Asian
56590016	chinese	33897005	Chinese	Asian
141631000000113	japanese - ethnic category 2001 census	92761000000103	Any other Asian background	Asian
286018012	south east asian	186044009	Any other Asian background	Asian
456650013	vietnamese	312859007	Any other Asian background	Asian
141651000000118	malaysian - ethnic category 2001 census	92781000000107	Any other Asian background	Asian
141641000000116	filipino - ethnic category 2001 census	92771000000105	Any other Asian background	Asian
141621000000111	vietnamese - ethnic category 2001 census	92751000000101	Any other Asian background	Asian
550541000006110	Chinese	33897005	Chinese	Asian
196661000006114	RACE: Japanese	414551003	Any other Asian background	Asian
196701000006118	RACE: Oriental	414978006	Any other Asian background	Asian
196671000006119	RACE: Korean	38361009	Any other Asian background	Asian
196651000006112	RACE: Chinese	33897005	Chinese	Asian
1564521000006118	Chinese	1564521000006102	Chinese	Asian
1564731000006113	Filipino	1564731000006109	Any other Asian background	Asian
1565011000006116	Japanese	1565011000006100	Any other Asian background	Asian
1565071000006113	Korean (North)	1565071000006109	Any other Asian background	Asian
1565081000006111	Korean (South)	1565081000006107	Any other Asian background	Asian
1565661000006112	Singaporean	1565661000006108	Any other Asian background	Asian

250230013	Far Eastern origin	160521004	Any other Asian background	Asian
250243013	race: west indian	160531006	Caribbean	Black
250231012	west indian origin	160522006	Caribbean	Black
1968361000006117	black/afri/carib/black brit: caribbean- ni eth cat 2011 cens	977811000000105	Caribbean	Black
1968551000006110	carib/black: caribbean/carib scot/carib brit- scotland 2011	978271000000103	Caribbean	Black
154401000000118	caribbean - ethnic category 2001 census	107691000000105	Caribbean	Black
5516681000006114	black caribbean/west india/guyana	270460000	Caribbean	Black
514611000006111	black caribbean	185988007	Caribbean	Black
1968191000006111	black/african/caribbn/black brit: caribbean - eng+wales 2011	976911000000101	Caribbean	Black
1564491000006115	central african	1564491000006104	African	Black
1968181000006113	black/african/carib/black brit: african- eng+wales 2011 cens	976891000000104	African	Black
285943014	black - other african country	185993005	African	Black
285971015	other african countries (nmo)	186010002	African	Black
4917081000006119	central african republic	223522008	African	Black
141391000000115	african - ethnic category 2001 census	92491000000104	African	Black
1968351000006119	black/afri/carib/black brit: african- ni eth cat 2011 census	977791000000109	African	Black
250223019	african origin	160514004	African	Black
1968531000006115	african: african/african scot/african brit - scotland 2011	978231000000100	African	Black
1565711000006118	south african	1565711000006102	African	Black
1968541000006113	african: any other african - scotland ethnic cat 2011 census	978251000000107	African	Black

30683015	black african	18167009	African	Black
453110019	black guyana	309644006	Caribbean	Black
459782019	other black ethnic group	315279003	Any other Black, Black British or Caribbean background	Black
158351000000119	other black background - ethnic category 2001 census	92501000000105	Any other Black, Black British or Caribbean background	Black
411580010	guyana (nmo)	275593008	Caribbean	Black
411578016	caribbean island (nmo)	275591005	Caribbean	Black
285948017	black indian sub-continent	185995003	Any other Black, Black British or Caribbean background	Black
285950013	black black - other	185989004	Any other Black, Black British or Caribbean background	Black
196601000006113	race: afro-caribbean	413465009	African	Black
285932018	black british	185990008	African	Black
453109012	black west indian	309643000	Caribbean	Black
411573013	black north african	275586009	African	Black
285931013	black, other, non-mixed origin	185989004	Any other Black, Black British or Caribbean background	Black
141591000000113	somali - ethnic category 2001 census	92711000000100	Any other Black, Black British or Caribbean background	Black
405067016	caribbean i./w.i./guyana (nmo)	270463003	Caribbean	Black
937731000006115	other black or black unspecified ethnic category 2001 census	92741000000104	Any other Black, Black British or Caribbean background	Black
141601000000119	nigerian - ethnic category 2001 census	92731000000108	African	Black
405064011	black caribbean/w.i./guyana	270460000	Caribbean	Black
1968571000006117	carib/black: any other black/caribbean grp - scotland 2011	978361000000101	Any other Black, Black British or Caribbean background	Black
285949013	black - other asian	185996002	Any other Black, Black British or	Black

			Caribbean background	
514651000006112	black e afric asia/indo-caribb	270462008	Any other Black, Black British or Caribbean background	Black
285930014	black caribbean	185988007	Caribbean	Black
411576017	black east african asian	275589002	Any other Black, Black British or Caribbean background	Black
459730016	black - ethnic group	315240009	African	Black
411577014	black indo-caribbean	275590006	Caribbean	Black
411575018	black iranian	275588005	Any other Black, Black British or Caribbean background	Black
157311000000119	black british - ethnic category 2001 census	110791000000100	African	Black
411579012	west indian (nmo)	275592003	Caribbean	Black
142811000000112	north african - ethnic category 2001 census	94061000000107	African	Black
1968371000006112	Black or African or Caribbean or Black British: other Black or African or Caribbean background - Northern Ireland ethnic category 2011 census	977831000000102	Any other Black, Black British or Caribbean background	Black
1968201000006114	Black or African or Caribbean or Black British: other Black or African or Caribbean background - England and Wales ethnic category 2011 census	976931000000109	Any other Black, Black British or Caribbean background	Black
1968561000006112	Caribbean or Black: Black, Black Scottish or Black British - Scotland ethnic category 2011 census	978341000000102	Any other Black, Black British or Caribbean background	Black
1564201000006114	Angolan	156420100000610 5	African	Black

1564801000006113	Ghanaian	1564801000006109	African	Black
1565651000006110	Sierra Leonean	1565651000006106	African	Black
1565701000006116	Somali	1565701000006100	African	Black
142781000000114	mixed irish and other white - ethnic category 2001 census	94021000000104	Any other Mixed or multiple ethnic background	Mixed
141321000000118	white and black caribbean - ethnic category 2001 census	92421000000102	White and Black Caribbean	Mixed
460153018	black caribbean and white	315634007	White and Black Caribbean	Mixed
1968091000006110	mixed: white+black caribbean - eng+wales eth cat 2011 census	976711000000103	White and Black Caribbean	Mixed
1968261000006110	mixed: white and black caribbean - ni ethnic cat 2011 census	977391000000108	White and Black Caribbean	Mixed
141331000000116	white and black african - ethnic category 2001 census	92431000000100	White and Black African	Mixed
460154012	black african and white	315635008	White and Black African	Mixed
1968101000006116	mixed: white+black african - eng+wales eth cat 2011 census	976731000000106	White and Black African	Mixed
196611000006111	race: afro-caucasian	413466005	White and Black African	Mixed
1968271000006115	mixed: white and black african - ni ethnic cat 2011 census	977411000000108	White and Black African	Mixed
141341000000113	white and asian - ethnic category 2001 census	92441000000109	White and Asian	Mixed
285991010	other ethnic, asian/white orig	186021006	White and Asian	Mixed
1968111000006118	mixed: white+asian - eng+wales ethnic category 2011 census	976751000000104	White and Asian	Mixed
1968281000006117	mixed: white and asian - ni ethnic	977431000000100	White and Asian	Mixed

	category 2011 census			
157291000000115	black and white - ethnic category 2001 census	110771000000104	Any other Mixed or multiple ethnic background	Mixed
285992015	other ethnic, mixed white orig	186022004	Any other Mixed or multiple ethnic background	Mixed
4740401000006118	other ethnic, other mixed origin	186023009	Any other Mixed or multiple ethnic background	Mixed
141471000000113	black and asian - ethnic category 2001 census	92581000000100	Any other Mixed or multiple ethnic background	Mixed
4740341000006112	other ethnic, black/white origin	186020007	Any other Mixed or multiple ethnic background	Mixed
937511000006119	other mixed or mixed unspecified ethnic category 2001 census	92621000000100	Any other Mixed or multiple ethnic background	Mixed
459729014	mixed ethnic census group	315239007	Any other Mixed or multiple ethnic background	Mixed
285993013	other ethnic, other mixed orig	186023009	Any other Mixed or multiple ethnic background	Mixed
1968291000006119	mixed: other mixed/multiple ethnic backgrd - ni 2011 census	977551000000106	Any other Mixed or multiple ethnic background	Mixed
285952017	other black - black/white orig	185999009	Any other Mixed or multiple ethnic background	Mixed
285990011	other ethnic, black/white orig	186020007	Any other Mixed or multiple ethnic background	Mixed
285953010	other black - black/asian orig	186000006	Any other Mixed or multiple ethnic background	Mixed
141481000000110	black and chinese - ethnic category 2001 census	92591000000103	Any other Mixed or multiple ethnic background	Mixed
285989019	other ethnic, mixed origin	186019001	Any other Mixed or multiple ethnic background	Mixed
1968471000006116	mixed/multiple ethnic grps: any-scot ethnic cat 2011 census	978051000000102	Any other Mixed or multiple ethnic background	Mixed

141491000000112	chinese and white - ethnic category 2001 census	92601000000109	White and Asian	Mixed
4740361000006111	other ethnic, asian/white origin	186021006	White and Asian	Mixed
158361000000116	asian and chinese - ethnic category 2001 census	92611000000106	White and Asian	Mixed
141351000000111	other mixed background - ethnic category 2001 census	92451000000107	Any other Mixed or multiple ethnic background	Mixed
4740381000006118	other ethnic, mixed white origin	186022004	Any other Mixed or multiple ethnic background	Mixed
141511000000116	mixed asian - ethnic category 2001 census	92631000000103	Any other Mixed or multiple ethnic background	Mixed
158371000000111	mixed black - ethnic category 2001 census	92721000000106	Any other Mixed or multiple ethnic background	Mixed
285951012	black - other, mixed	185998001	Any other Mixed or multiple ethnic background	Mixed
1968121000006114	Mixed multiple ethnic groups: any other Mixed or multiple ethnic background - England and Wales ethnic category 2011 census	976771000000108	Any other Mixed or multiple ethnic background	Mixed
196681000006116	RACE: Mixed	414752008	White and Black Caribbean	Mixed
713341000000111	british israelite	368051000000100	Any other ethnic group	Other
411574019	black arab	275587000	Arab	Other
405065012	black n african/arab/iranian	270461001	Arab	Other
1968581000006119	other ethnic grp: arab/arab scot/arab british- scotland 2011	978381000000105	Arab	Other
1968211000006112	other ethnic group: arab - eng+wales ethnic cat 2011 census	976951000000102	Arab	Other
4740241000006117	british ethnic minority specified (nmo)	186006000	Any other ethnic group	Other

411581014	north african arab (nmo)	275594002	Arab	Other
1968591000006116	other ethnic grp: any other ethnic grp- scotland 2011 census	978401000000105	Any other ethnic group	Other
138261000000114	iranian - ethnic category 2001 census	89011000000107	Any other ethnic group	Other
286022019	new zealand ethnic group nos	186035008	Any other ethnic group	Other
937871000006114	mid east (excl israeli, iranian & arab) - eth cat 2001 cens	94071000000100	Any other ethnic group	Other
141411000000115	other - ethnic category 2001 census	92521000000101	Any other ethnic group	Other
286017019	other pacific ethnic group	372148003	Any other ethnic group	Other
138241000000113	jewish - ethnic category 2001 census	88991000000105	Any other ethnic group	Other
142901000000119	any other group - ethnic category 2001 census	94151000000105	Any other ethnic group	Other
285959014	brit. ethnic minor. spec.(nmo)	186006000	Any other ethnic group	Other
1968221000006116	other ethnic: any other grp - eng+wales eth cat 2011 census	976971000000106	Any other ethnic group	Other
142881000000117	buddhist - ethnic category 2001 census	94131000000103	Any other ethnic group	Other
1968391000006113	other ethnic group: any other grp- ni ethnic cat 2011 census	977871000000100	Any other ethnic group	Other
937941000006111	mauritian/seychellois/maldivian/st helena eth cat 2001census	94121000000100	Any other ethnic group	Other
1968381000006110	other ethnic group: arab - ni ethnic category 2011 census	977851000000109	Arab	Other
405068014	n african arab/iranian (nmo)	270464009	Arab	Other
138271000000119	south and central american - ethnic	89021000000101	Any other ethnic group	Other

	category 2001 census			
285988010	other ethnic nec (nmo)	186005001	Any other ethnic group	Other
142841000000113	kurdish - ethnic category 2001 census	94091000000101	Any other ethnic group	Other
138251000000111	arab - ethnic category 2001 census	89001000000105	Arab	Other
4740261000006118	british ethnic minority unspecified (nmo)	186007009	Any other ethnic group	Other
142851000000111	moroccan - ethnic category 2001 census	94101000000109	Arab	Other
411582019	iranian (nmo)	275595001	Any other ethnic group	Other
142891000000115	sikh - ethnic category 2001 census	94141000000107	Any other ethnic group	Other
142861000000114	latin american - ethnic category 2001 census	94111000000106	Any other ethnic group	Other
142831000000116	israeli - ethnic category 2001 census	94081000000103	Any other ethnic group	Other
138281000000117	muslim - ethnic category 2001 census	89031000000104	Any other ethnic group	Other
286021014	other new zealand ethnic group	186035008	Any other ethnic group	Other
285958018	other ethnic non-mixed (nmo)	186005001	Any other ethnic group	Other
459785017	other ethnic group	372148003	Any other ethnic group	Other
285960016	brit. ethnic minor. unsp (nmo)	186007009	Any other ethnic group	Other
157351000000115	hindu - ethnic category 2001 census	110831000000107	Any other ethnic group	Other
286006015	new zealand ethnic groups	186035008	Any other ethnic group	Other
1572831000000110	Nepali	718131000000106	Any other ethnic group	Other
286012013	New Zealand Maori	186039002	Any other ethnic group	Other
507015012	Samoan	86275006	Any other ethnic group	Other
286013015	Cook Island Maori	186040000	Any other ethnic group	Other

504723011	Tongan	81560001	Any other ethnic group	Other
286014014	Niuean	186041001	Any other ethnic group	Other
286015010	Tokelauan	186042008	Any other ethnic group	Other
501416013	Fijian	69865008	Any other ethnic group	Other
523591000000116	Yemeni	296841000000102	Arab	Other
196621000006115	RACE: Arab	90027003	Arab	Other
371005013	RACE: Other	103579009	Any other ethnic group	Other
250225014	North American origin	160516002	Any other ethnic group	Other
250226010	South American origin	160517006	Any other ethnic group	Other
1564151000006117	Afghan	1564151000006101	Any other ethnic group	Other
1564181000006113	American	1564181000006109	Any other ethnic group	Other
1564941000006117	Iranian	1564941000006101	Any other ethnic group	Other
1564951000006115	Iraqi	1564951000006104	Arab	Other
1565131000006111	Lebanese	1565131000006107	Arab	Other
1565151000006116	Libyan	1565151000006100	Arab	Other
1565321000006110	Moroccan	1565321000006106	Arab	Other
1565691000006116	Solomon Islander	1565691000006100	Any other ethnic group	Other
250227018	Australian origin	160518001	Any other ethnic group	Other
250229015	Middle Eastern origin	160520003	Any other ethnic group	Other
781081000006113	indian	414481008	Indian	South Asian
1968131000006112	asian/asian brit: indian - eng+wales ethnic cat 2011 census	976791000000107	Indian	South Asian
1968301000006118	asian or asian british: indian - ni ethnic cat 2011 census	977591000000103	Indian	South Asian
157271000000119	indian or british indian - ethnic category 2001 census	110751000000108	Indian	South Asian

1564921000006112	indian	156492100000610 8	Indian	South Asian
285954016	indian	414481008	Indian	South Asian
1968491000006115	asian: indian, indian scot/indian brit-scotland 2011 census	978111000000100	Indian	South Asian
250228011	indian origin	160519009	Indian	South Asian
1968141000006119	asian/asian british:pakistani-eng+wales eth cat 2011 census	976811000000108	Pakistani	South Asian
141361000000114	pakistani or british pakistani - ethnic category 2001 census	92461000000105	Pakistani	South Asian
285955015	pakistani	186002003	Pakistani	South Asian
1565441000006111	pakistani	156544100000610 7	Pakistani	South Asian
1968311000006115	asian/asian british: pakistani - ni ethnic cat 2011 census	977711000000100	Pakistani	South Asian
196721000006111	race: pakistani	186002003	Pakistani	South Asian
1968481000006118	asian: pakistani/pakistani scot/pakistani brit-scot 2011	978071000000106	Pakistani	South Asian
1564291000006119	bangladeshi	156429100000610 3	Bangladeshi	South Asian
1968501000006111	bangladeshi, bangladeshi scot or bangladeshi brit-scot 2011	978171000000105	Bangladeshi	South Asian
196631000006117	race: bangladeshi	186003008	Bangladeshi	South Asian
285956019	bangladeshi	186003008	Bangladeshi	South Asian
1968321000006111	asian/asian british: bangladeshi - ni ethnic cat 2011 census	977731000000108	Bangladeshi	South Asian
937541000006115	bangladeshi or british bangladeshi - ethn categ 2001 census	92471000000103	Bangladeshi	South Asian
1968151000006117	asian/asian brit: bangladeshi-	976831000000100	Bangladeshi	South Asian

	eng+wales eth cat 2011 census			
250224013	asian origin	160515003	Indian	South Asian
141561000000119	british asian - ethnic category 2001 census	92681000000104	Indian	South Asian
412016016	o/e - asian origin	276029002	Indian	South Asian
141431000000111	scottish - ethnic category 2001 census	92541000000108	English, Welsh, Scottish, Northern Irish or British	White
2486161000000112	white - northern ireland ethnic category 2011 census	977351000000100	English, Welsh, Scottish, Northern Irish or British	White
141451000000116	northern irish - ethnic category 2001 census	92561000000109	English, Welsh, Scottish, Northern Irish or British	White
1780408016	other white british ethnic group	401214002	English, Welsh, Scottish, Northern Irish or British	White
2487321000000116	white: other british - scotland ethnic category 2011 census	977931000000106	English, Welsh, Scottish, Northern Irish or British	White
1564391000006113	british	1564391000006109	English, Welsh, Scottish, Northern Irish or British	White
1968051000006116	white:eng/welsh/scot/ni/brit - england and wales 2011 census	976631000000101	English, Welsh, Scottish, Northern Irish or British	White
158341000000117	british or mixed british - ethnic category 2001 census	92391000000108	English, Welsh, Scottish, Northern Irish or British	White
459726019	white british	315236000	English, Welsh, Scottish, Northern Irish or British	White
196641000006110	race: caucasian	413773004	English, Welsh, Scottish, Northern Irish or British	White
1780407014	white scottish	401213008	English, Welsh, Scottish, Northern Irish or British	White
1063981000000117	white british - ethnic category 2001 census	494131000000105	English, Welsh, Scottish, Northern Irish or British	White

2487281000000112	white: scottish - scotland ethnic category 2011 census	977911000000103	English, Welsh, Scottish, Northern Irish or British	White
157281000000117	english - ethnic category 2001 census	110761000000106	English, Welsh, Scottish, Northern Irish or British	White
142691000000116	ulster scots - ethnic category 2001 census	93921000000101	English, Welsh, Scottish, Northern Irish or British	White
6846371000006111	caucasian	413773004	English, Welsh, Scottish, Northern Irish or British	White
285925010	white	185984009	English, Welsh, Scottish, Northern Irish or British	White
141441000000119	welsh - ethnic category 2001 census	92551000000106	English, Welsh, Scottish, Northern Irish or British	White
141461000000118	cornish - ethnic category 2001 census	92571000000102	English, Welsh, Scottish, Northern Irish or British	White
2484671000000118	white: irish - england and wales ethnic category 2011 census	976651000000108	Irish	White
2487361000000112	white: irish - scotland ethnic category 2011 census	977951000000104	Irish	White
1564961000006118	irish	156496100000006102	Irish	White
285978014	irish (nmo)	186014006	Irish	White
1064041000000111	white irish - ethnic category 2001 census	494161000000100	Irish	White
141301000000110	irish - ethnic category 2001 census	92401000000106	Irish	White
459727011	white irish	315237009	Irish	White
937411000006115	other white or white unspecified ethnic category 2001 census	94051000000109	Any other White background	White
937311000006113	commonwealth (russian) indep states - ethn categ 2001 census	88961000000104	Any other White background	White
138201000000110	polish - ethnic category 2001 census	88941000000100	Any other White background	White

142791000000111	other mixed white - ethnic category 2001 census	94031000000102	Any other White background	White
138191000000113	gypsy/romany - ethnic category 2001 census	88931000000109	Roma	White
138231000000116	albanian - ethnic category 2001 census	88971000000106	Any other White background	White
459728018	other white ethnic group	185984009	Any other White background	White
142741000000118	kosovan - ethnic category 2001 census	93981000000100	Any other White background	White
285987017	other european (nmo)	186017004	Any other White background	White
157991000000110	serbian - ethnic category 2001 census	88981000000108	Any other White background	White
411594011	greek (nmo)	275599007	Any other White background	White
937371000006116	other republics former yugoslavia - ethnic categ 2001 census	94011000000105	Any other White background	White
1968251000006113	irish traveller - northern ireland ethnic cat 2011 census	977371000000109	Gypsy or Irish Traveller	White
138171000000114	irish traveller - ethnic category 2001 census	88911000000101	Gypsy or Irish Traveller	White
142721000000113	turkish cypriot - ethnic category 2001 census	93951000000106	Any other White background	White
1968081000006112	white: other white backgrd- eng+wales ethnic cat 2011 census	976691000000100	Any other White background	White
142751000000115	bosnian - ethnic category 2001 census	93991000000103	Any other White background	White
2645811000000115	roma ethnic group	718958002	Roma	White
937391000006115	oth white european/european unsp/mixed european 2001 census	94041000000106	Any other White background	White

1968461000006111	white: other white ethnic grp- scotland ethnic cat 2011 cens	978031000000109	Any other White background	White
156921000000110	turkish - ethnic category 2001 census	110401000000103	Any other White background	White
141661000000115	cypriot (part not stated) - ethnic category 2001 census	92791000000109	Any other White background	White
411595012	greek cypriot (nmo)	275600005	Any other White background	White
142761000000117	croatian - ethnic category 2001 census	94001000000108	Any other White background	White
937301000006110	baltic estonian/latvian/lithuanian - ethn categ 2001 census	88951000000102	Any other White background	White
141311000000112	other white background - ethnic category 2001 census	92411000000108	Any other White background	White
138181000000111	traveller - ethnic category 2001 census	88921000000107	Gypsy or Irish Traveller	White
158481000000115	italian - ethnic category 2001 census	93961000000109	Any other White background	White
142711000000119	greek cypriot - ethnic category 2001 census	93941000000108	Any other White background	White
1968441000006112	white: gypsy/irish traveller - scotland ethnic cat 2011 cens	977971000000108	Gypsy or Irish Traveller	White
142701000000116	greek - ethnic category 2001 census	93931000000104	Any other White background	White
411597016	turkish cypriot (nmo)	275602002	Any other White background	White
2487481000000113	white: polish - scotland ethnic category 2011 census	978011000000101	Any other White background	White
459786016	irish traveller	315283003	Gypsy or Irish Traveller	White
405071018	turkish/turkish cypriot (nmo)	270467002	Any other White background	White
405070017	greek/greek cypriot (nmo)	270466006	Any other White background	White

411596013	turkish (nmo)	275601009	Any other White background	White
1968071000006114	white: gypsy/irish traveller - eng+wales eth cat 2011 census	976671000000104	Gypsy or Irish Traveller	White
286007012	New Zealand European	186036009	Any other White background	White
1551471000000116	Slovak	36329002	Any other White background	White
286008019	Pakeha	186036009	Any other White background	White
286009010	Other European in New Zealand	186037000	Any other White background	White
850671000006119	Traveller - gypsy	40182006	Gypsy or Irish Traveller	White
2537217015	RACE: White	413773004	English, Welsh, Scottish, Northern Irish or British	White
133078012	Portuguese	80208004	Any other White background	White
250222012	European origin	160513005	Any other White background	White
1564161000006115	Albanian	1564161000006104	Any other White background	White
1564811000006111	Greek	1564811000006107	Any other White background	White
1564881000006116	Herzegovinian	1564881000006100	Any other White background	White
1564981000006111	Italian	1564981000006107	Any other White background	White
1565511000006113	Portuguese	1565511000006109	Any other White background	White
1573201000006119	Portuguese	1573201000006103	Any other White background	White
2615361000000115	Slovak Roma	718961001	Roma	White
2615461000000111	Czech Roma	718959005	Roma	White
2615531000000117	Hungarian Roma	718963003	Roma	White
2615571000000115	Polish Roma	718964009	Roma	White
2615611000000112	Romanian Roma	718960000	Roma	White
2615691000000115	Bulgarian Roma	718962008	Roma	White
7779801000006116	Roma	718958002	Roma	White
1158211000000111	Romanian	445343003	Any other White background	White

1158301000000115	Bulgarian	29343004	Any other White background	White
1160331000000119	Czech	286009	Any other White background	White
6846391000006112	Caucasoid race	413773004	English, Welsh, Scottish, Northern Irish or British	White
